# Supplementary material for: Spatiotemporally Asymmetric Excitation Supports Mammalian Retinal Motion Sensitivity
Source: Curr Biol. 2019 Oct 7;29(19):3277–3288.e5. doi: 10.1016/j.cub.2019.08.048 (PMC6865067; doi:10.1016/j.cub.2019.08.048)
Supplement: Document S1. Figures S1–S7 [file mmc1.pdf]

Current Biology, Volume *29*

Supplemental Information

Spatiotemporally Asymmetric Excitation Supports Mammalian Retinal Motion Sensitivity

Akihiro Matsumoto, Kevin L. Briggman, and Keisuke Yonehara

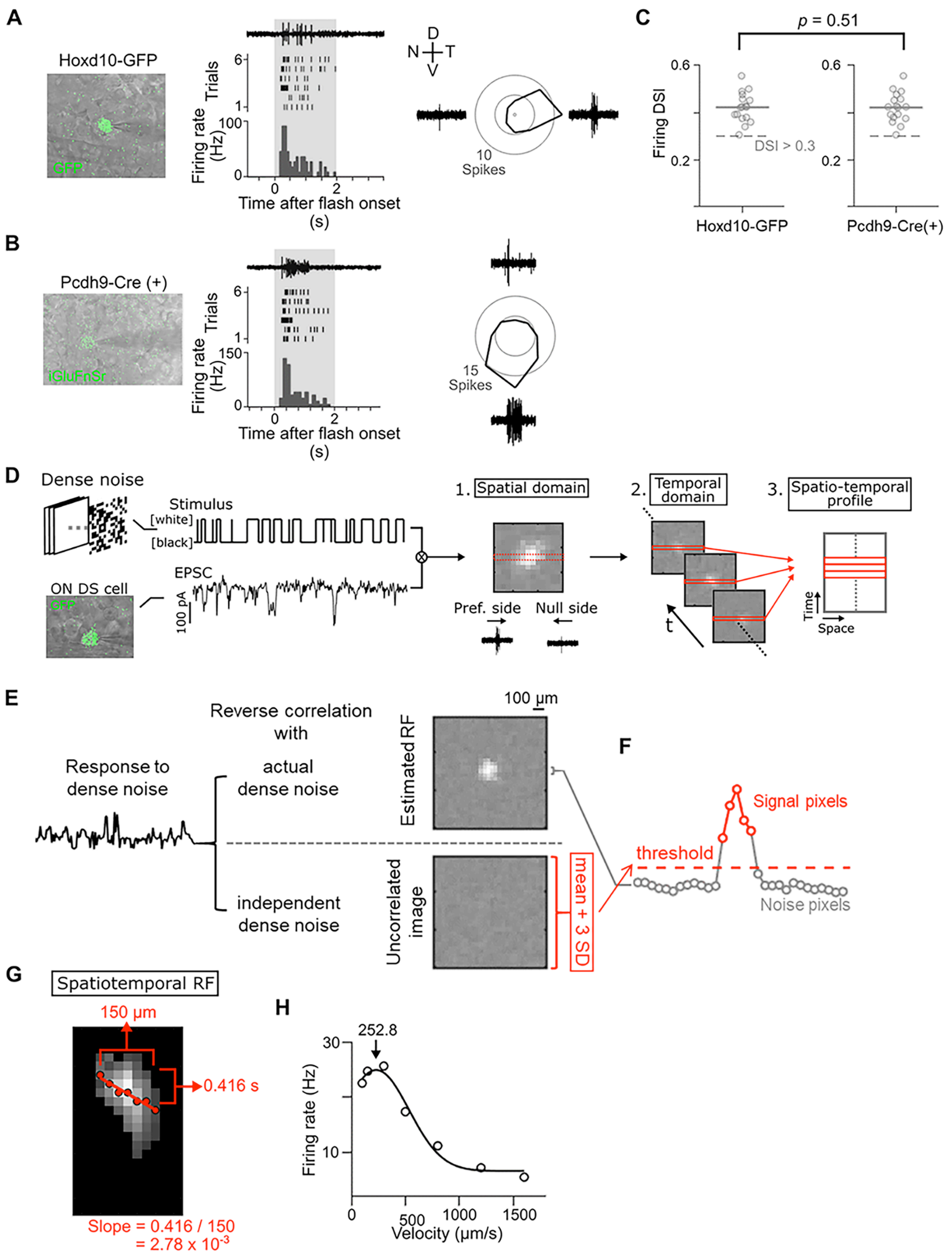

Figure S1. Recordings of Excitatory Inputs to Genetically-Labeled ON DS Cells, Related to Figure 1.

(A) Spike recording from an example Hoxd10-EGFP-labeled ganglion cell. It is known that all subtypes of ON DS cells are genetically labeled in Hoxd10-GFP mice. **(B)** Spike recording from

an example Pcdh9-Cre-labeled ganglion cell. It is known that ventrally-tuned ON DS cell subtype is genetically labeled in Pcdh9-Cre mice. **(C)** Firing DSI to a moving spot (300 μm in diameter, 150 μm/s) in Hoxd10-GFP and Pcdh9-Cre positive cells. All cells showed DS firings with more than 0.3 DSI. There were no significant differences in DSI between Hoxd10-GFP (17 cells) and Pcdh9-Cre positive cells (17 cells). $p = 0.51$, Mann-Whitney-Wilcoxon [MWW] test). **(D)** Top, reverse correlation using dense noise stimulus and evoked EPSC. Bottom, 1) spatial domain of RF. Red rectangle, a row including RF center along preferred-null axis. 2) temporal domain of RF at different synaptic delays was collected, and 3) aligned in spatiotemporal profile. **(E and F)** To make a criteria to determine a threshold for receptive field, we performed reverse correlation using response (EPSC or glutamate signal) to dense noise and independent dense noise ("uncorrelated image" in E). We calculated mean + 3 SD (red dashed line in F) of the uncorrelated image as a threshold to detect signal pixels in spatial receptive field (red circles in F). **(G)** Signal pixels in a thresholded spatiotemporal RF. Peak times in each spatial dimension (red circles) were fitted by linear regression (red line). The slope in peak time was calculated based on the fitted line (e.g. 0.418 s / 150 μm). **(H)** The optimal velocity in firings was calculated by a parameter (arrow) in the fitted Gaussian function.

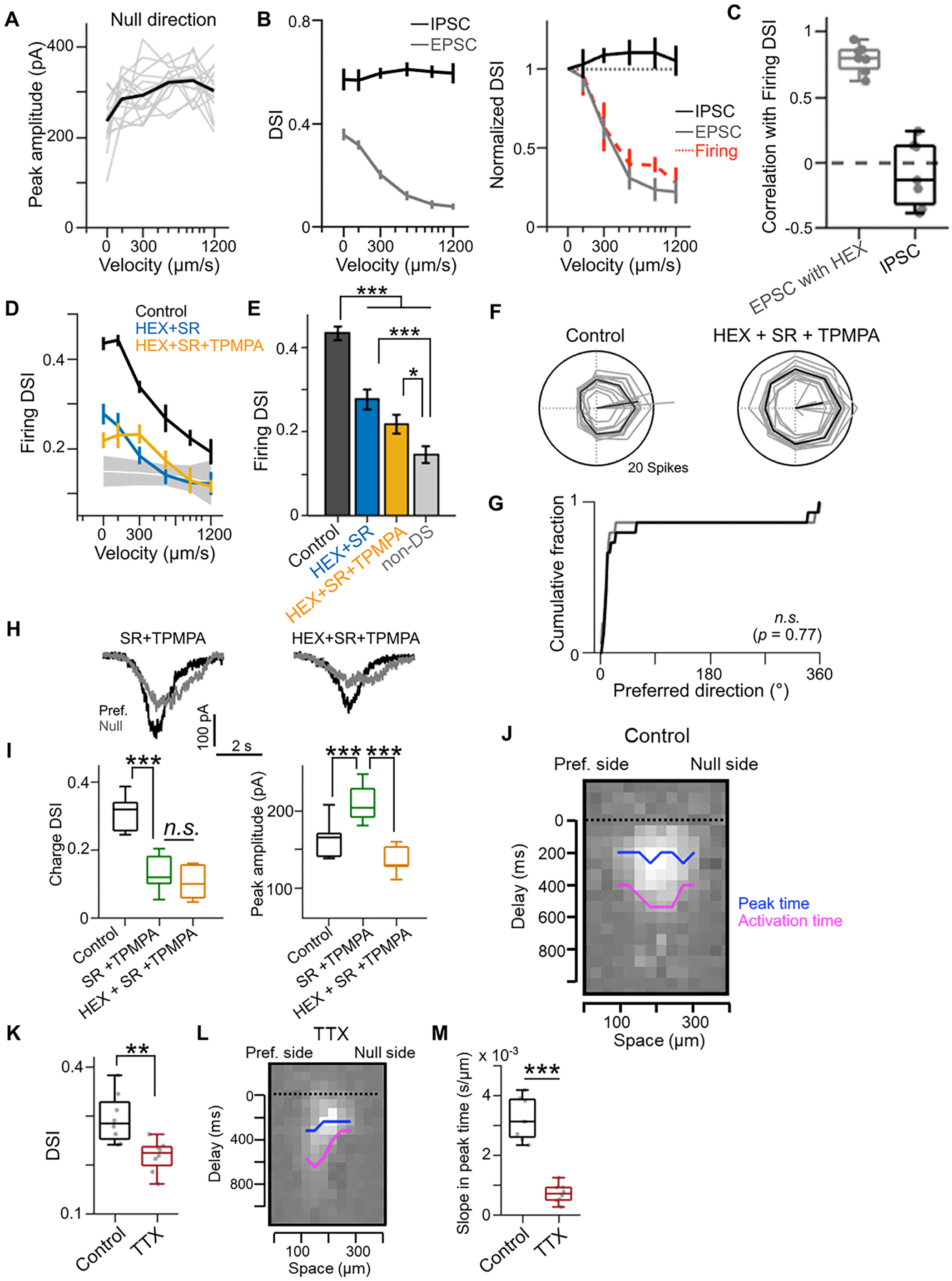

Figure S2. Electrophysiological Characterization of Inhibitory Inputs to ON DS Cells, Related to Figure 1.

(A) Inhibitory postsynaptic current (IPSC) amplitude recorded from ON DS cells as a function of stimulus velocity in null direction. Thin line, individual cell. Thick line, average. **(B)** Left, DSI calculated from peak amplitude of EPSC (gray) and IPSC (black). Right, DSI normalized to that

at 150 µm/s. 7 cells. Error bar, SE. **(C)** Correlation of ESPC and IPSC with firing DSI. **(D)** Relationship between firing DSI and motion velocity. Black, control, 16 ON DS cells. Blue, 2 µM hexamethonium (HEX) + 50 µM SR95531 (SR), 13 ON DS cells. Orange, HEX + SR + 100 µM TPMPA, 8 ON DS cells. Gray shaded, 95 % CI calculated from 16 non-DS cells. **(E)** Firing DSI to motion stimulus in 150 µm/s. Mann-Whitney-Wilcoxon test. **(F)** Directional tunings of firings in control (left) and HEX+SR+TPMPA (right). Gray, individual cells. Black, average. **(G)** Cumulative histogram of preferred direction (F). Gray, control. Black, HEX+SR+TPMPA. 12 cells. Kolmogorov-Smirnov test. **(H)** EPSCs to preferred- (black) and null- (gray) direction motion in SR+TPMPA (left) and in HEX+SR+TPMPA (right). **(I)** Charge DSI (left) and peak amplitude (right) of EPSC in control, SR+TPMPA (green) and HEX+SR+TPMPA (orange). 6 cells. Mann-Whitney-Wilcoxon test. **(J)** Spatiotemporal receptive field (stRF) for IPSC recorded from an ON DS cell. Detected peak timing (blue) and activation timing (magenta) were overlaid. Dotted black, timing of synaptic input. **(K)** DSI of EPSC to 150 µm/s motion in control (black) and in blocking of Na_V (TTX, 1 µM, dark red). 8 cells. One-tailed Wilcoxon signed-rank sum test. **(L)** Excitatory stRF in TTX. (M) Slope in peak time in control (black) and TTX (dark red). 8 cells. One-tailed Wilcoxon signed-rank sum test. *, $p < 0.05$. **, $p < 0.01$. ***, $p < 0.001$.

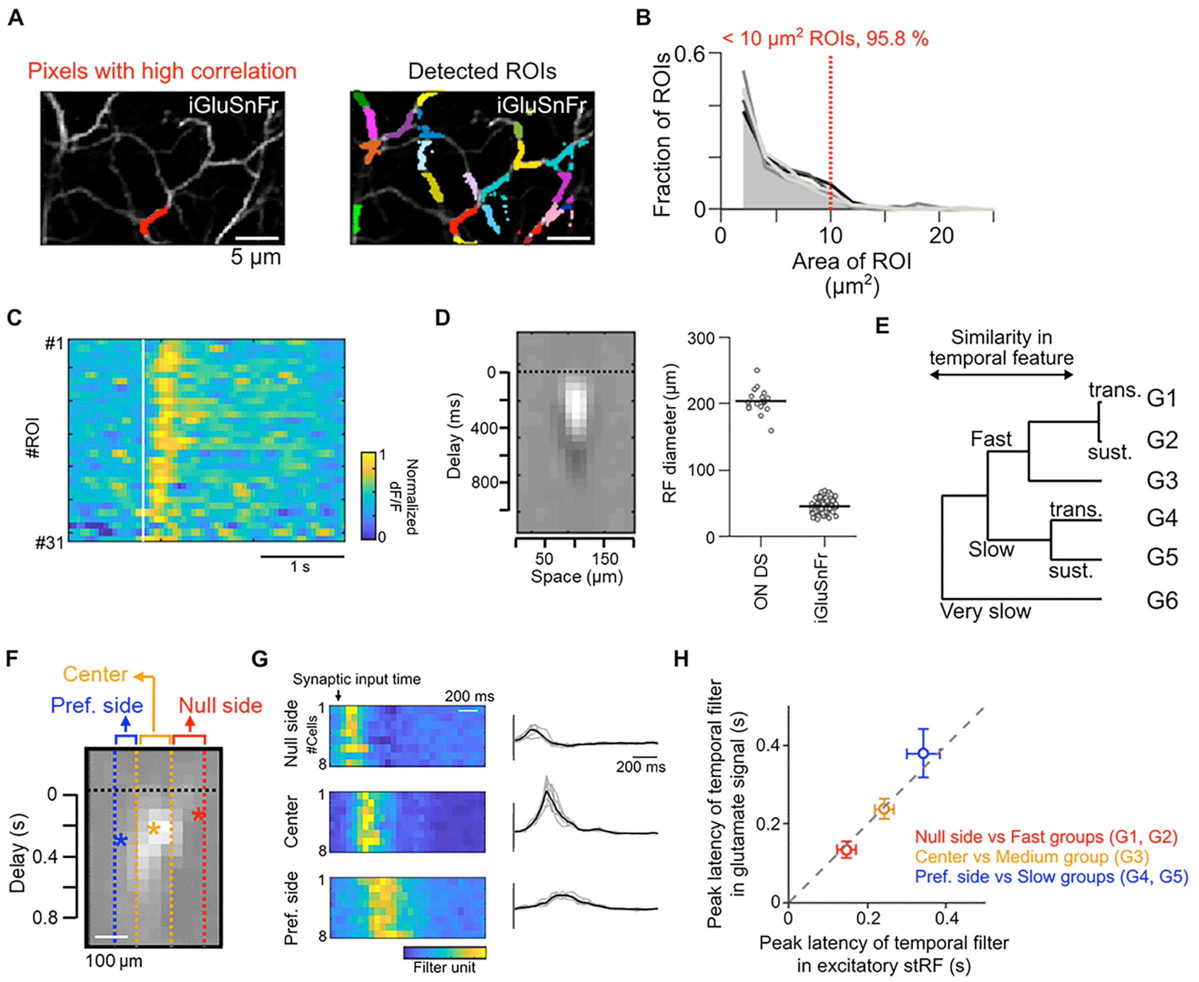

Figure S3. Determination of ROIs on the Dendrites of Genetically Labeled ON DS Cells for Glutamate Imaging, Related to Figure 2.

(A) Left, two-photon image of ON DS cell dendrites expressing iGluSnFr. Pixels showing high temporal correlation (> 0.4) in response to static flash stimulus were assigned to the same ROI (red pixels). Right, detected ROIs indicated by distinct colors. **(B)** Distribution of the area of ROIs. Based on the size of axonal boutons determined by a previous anatomical and glutamate imaging study (see ref. Franke et al., 2017), we set an upper limit of the area size to 10 μm^2. 95.8 % of responsive ROIs met this criterion. **(C)** Responses of ROIs detected in one field of view to a static flash for 2 s. White line, stimulus onset. **(D)** Left, an example stRF of glutamate signal. Spatiotemporally asymmetric structure is not obvious. Right, RF diameter of ON DS cells (17 cells) estimated by firings recorded by cell-attached recordings and spatial RF diameter of individual ROIs of glutamate signal (iGluSnFr, 128 ROIs). **(E)** Dendrogram estimated by

hierarchical clustering using temporal features: peak latency, decay, preferred frequency and contrast, correlation between glutamate signal and stimulus profile. **(F)** Schematic for the definition of the the preferred, center, and null side stRF (see also STAR Methods). We first detected three pixels: a center pixel (orange star) showing the maximum filter unit, and two pixels at the most preferred (blue star) and null (red star) side stRF. The center stRF (flanked by orange dotted lines) was defined as the three columns in a way that the central column includes the center pixel (orange star). The preferred and null side stRFs were defined as the columns between the blue or red star and the orange dotted line, respectively. **(G)** Left, heatmaps showing the calculated temporal filters at null side (top), center (middle), and preferred side (bottom) in 8 cells. Right, line plots of temporal filters in individual (gray) and the average (balck). Gray horizontal line, time of synaptic inputs. **(H)** Relationship between peak latency of temporal filters in excitatory stRF (red, null side; orange, center; blue, preferred side) and peak latency of temporal filter in glutamate signal (red, fast groups; orange, medium group; blue, slow groups). Mean ± SE in 8 cells.

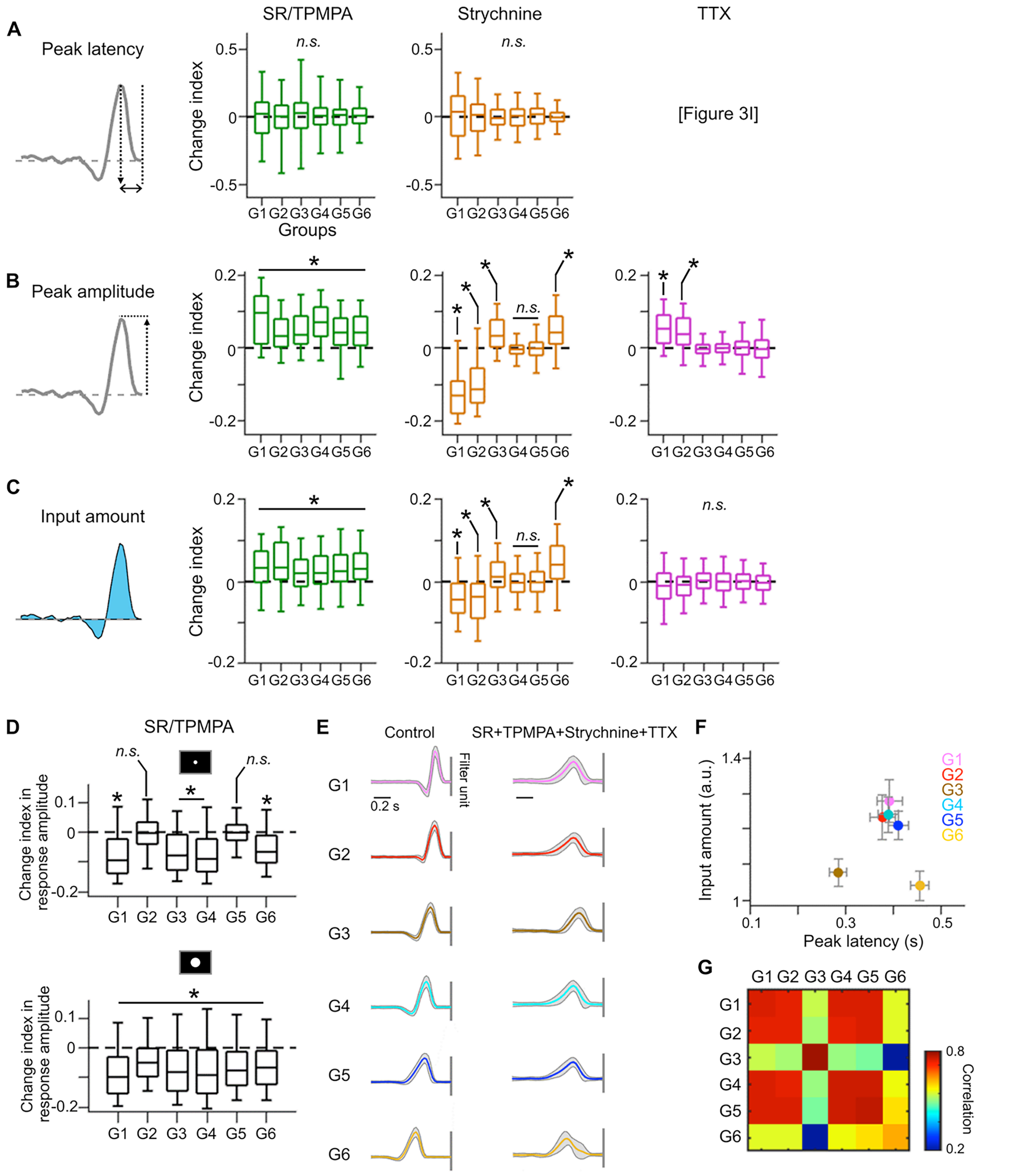

Figure S4. Different Effects of Pharmacological Blocking on Filter Properties of Glutamatergic Inputs, Related to Figures 3 and 4.

Effects of pharmacological blocking on temporal RFs were quantified by change index (see STAR Methods). Positive and negative values indicate increases and decreases in parameters by

application of blockers, respectively. Green, SR+TPMPA (50 and 100 µM). Gold, strychnine (1 µM). Purple, TTX (1 µM). **(A)** Change index in peak latency. Effects of TTX were shown in Figure 3I. **(B)** Change index in peak amplitude.**(C)** Change index in input amounts. **(D)** Change index in response amplitude of glutamate signals to small (top, 50 µm diameter) and large (bottom, 500 µm diameter) static flashing spot. **(E)** Temporal filter in control (left) and after the blocking of GABA, glycine, and acetylcholine receptors (right). Colored line, average. Gray shade, SD. **(F)** Peak latency and input amount of temporal filters in SR+TPMPA+Strychnine+HEX. Mean ± SE. **(G)** Mean correlation in the shape of temporal filters among the six groups. *, $p < 0.001$. One-tailed Wilcoxon signed-rank test.

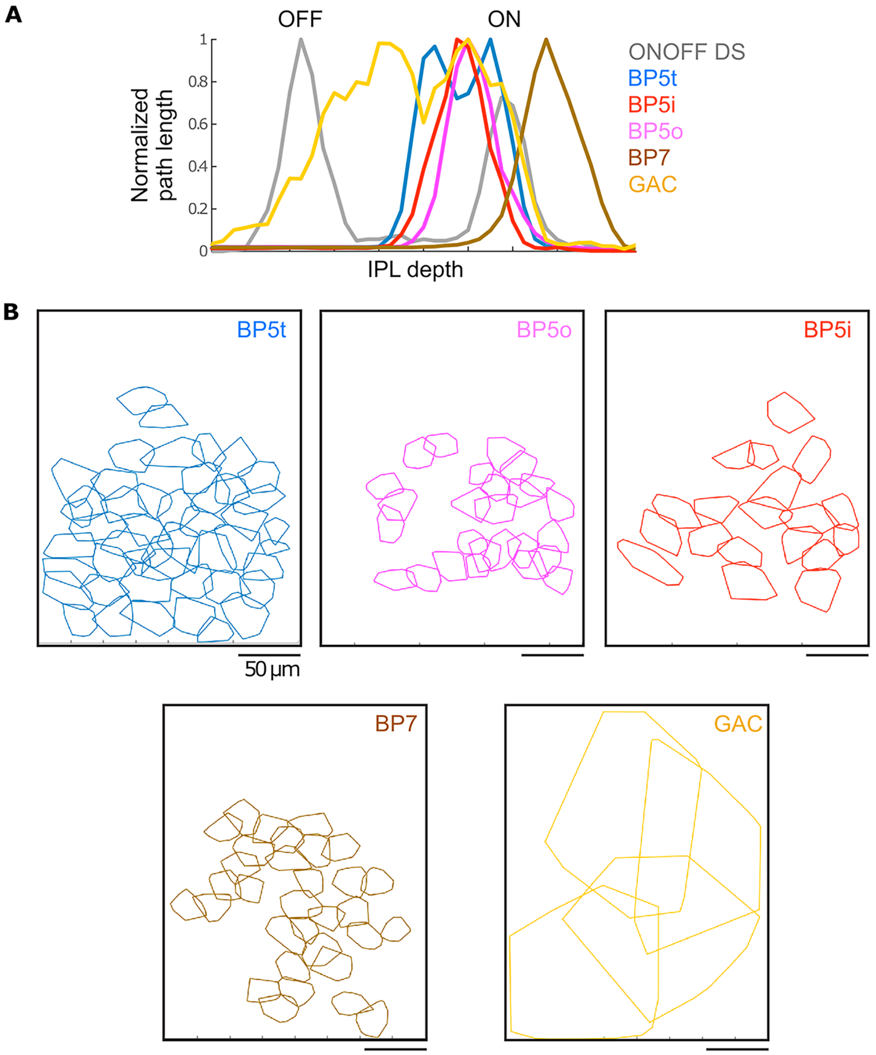

Figure S5. Classification of Excitatory Cell Types Presynaptic to ON DS Cells, Related to Figures 5 and 6.

(A) Stratification profiles of GACs and bipolar (BP) cells relative to OFF and ON DS cell bands in the IPL. **(B)** Mosaic of axonal convex hulls of BP types 5t, 5i, 5o, 7 and GACs. Scale bars: 50 µm.

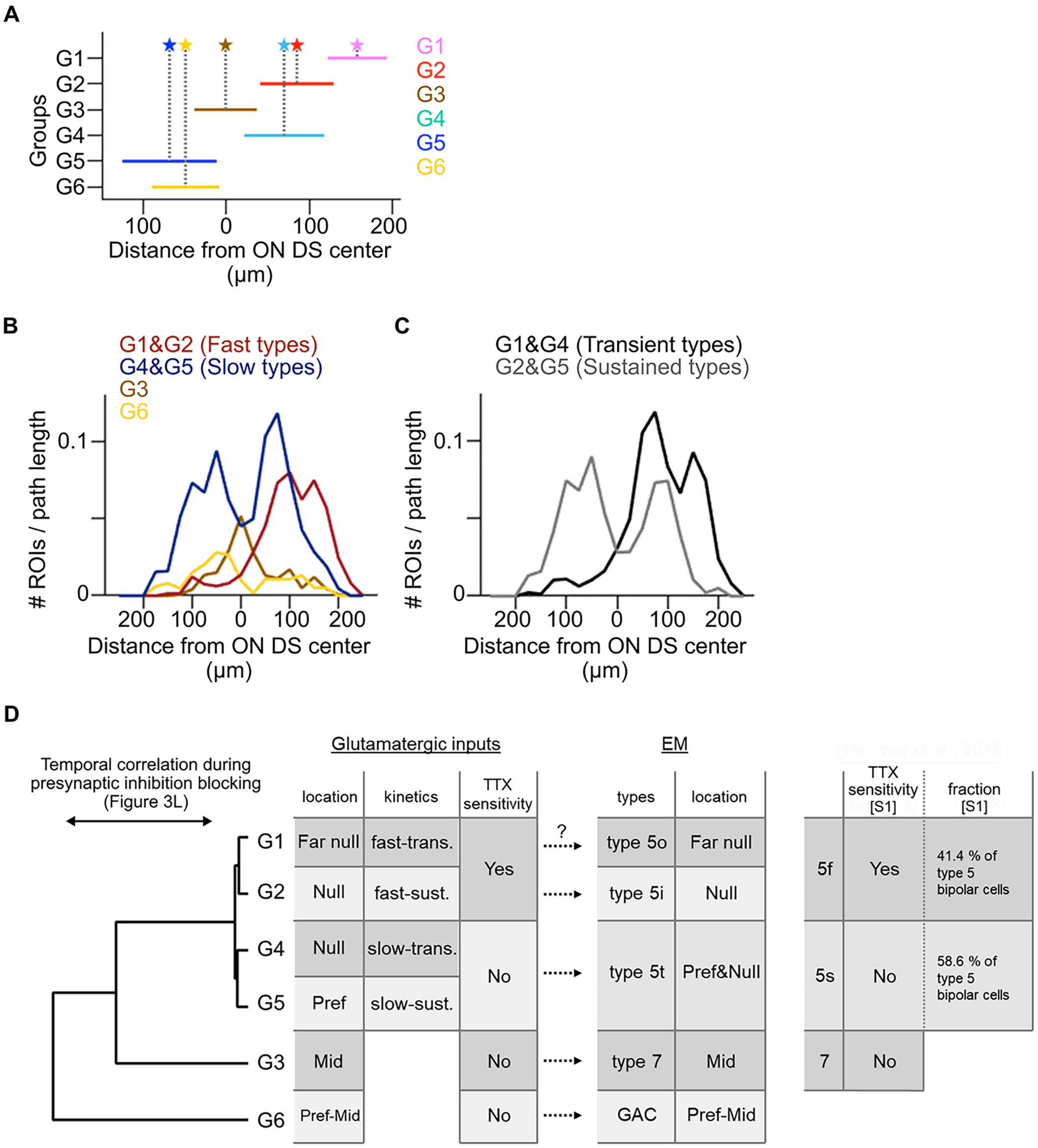

Figure S6. Spatial Distribution of Glutamatergic Input Groups, Related to Figures 5 and 6. **(A)** Summary of input location of the six glutamatergic groups. Each histogram of input location (Figure 5) was fitted by Gaussian distribution; mean (stars) and SD (horizontal line) in the fitted distribution were used to determine the input location. **(B and C)** Histograms of input location normalized by path length. Distributions of fast (G1 and G2) versus slow (G4 and G5) were summated in (B), and transient (G1 and G4) versus sustained (G2 and G5) were summated in (C). These distributions show that fast and transient inputs are biased to null side of ON DS cell dendrites. **(D)** Summaries for temporal correlation (dendrogram left), glutamatergic inputs (left

table), EM connectomics (center table), showing an estimation of the correspondence of the six groups in glutamate imaging to anatomical subtypes, and a reference of TTX sensitivity in anatomical subtypes from [S1] (right table).

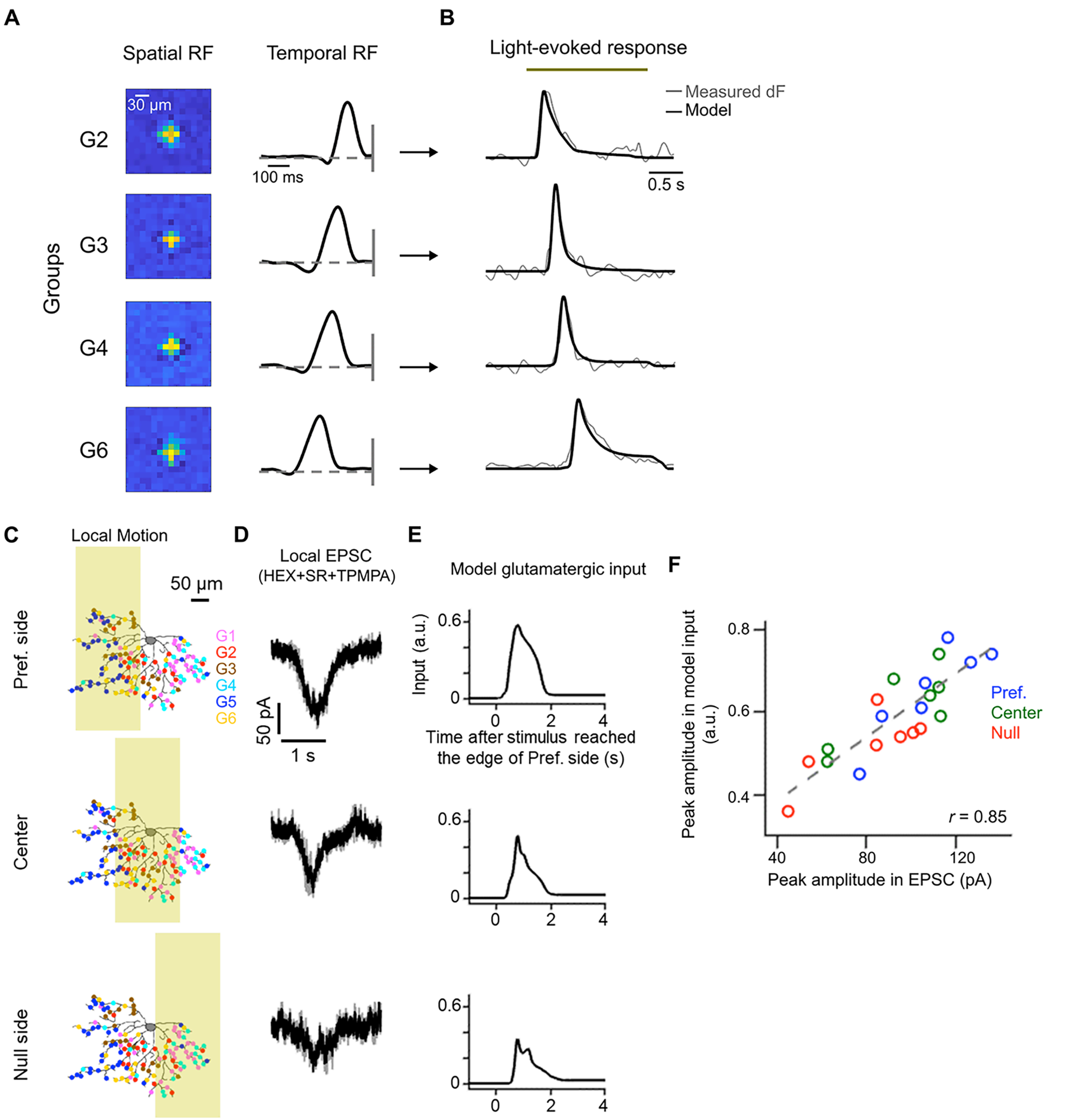

Figure S7. Linear Receptive Field Model to Simulate Light-Evoked Responses, Related to Figure 7.

(A) Spatial (left) and temporal (right) RFs in example 4 ROIs of G2, G3, G4, and G6 groups. Examples of G1 and G5 ROIs were shown in Figure 7A. **(B)** Simulated glutamatergic inputs to static flash (yellow horizontal bar) in the ROIs in (A). Gray, measured glutamatergic inputs. Black, modeled inputs. **(C)** Schematic for local motion (yellow band, 150 µm width × 400 µm length, 300 µm/s) at the preferred side (top), center (middle), and null side (bottom). **(D)** EPSCs to preferred-direction local motion at the preferred (top), center (middle), and null side (bottom). Gray, each trial. Black, average. **(E)** Model glutamatergic inputs simulated using ROIs which

were included in each local part (yellow bands in Figure S7C). **(F)** Relationship between peak amplitude in EPSC and model glutamatergic inputs at the preferred side (blue), center (green), and null side (red). 8 cells. $r = 0.85$. $p < 0.001$.

Supplemental Reference

S1. Hellmer, C.B., Zhou, Y., Fyk-Kolodziej, B., Hu, Z., and Ichinose, T. (2016). Morphological and physiological analysis of type-5 and other bipolar cells in the Mouse Retina. Neuroscience *315*, 246-258.
