# Supplementary material for: Spatiotemporally Asymmetric Excitation Supports Mammalian Retinal Motion Sensitivity
Source: Curr Biol. 2019 Oct 7;29(19):3277–3288.e5. doi: 10.1016/j.cub.2019.08.048 (PMC6865067; doi:10.1016/j.cub.2019.08.048)
Supplement: Document S2. Article plus Supplemental Information [file mmc2.pdf]

Article
Current Biology

Spatiotemporally Asymmetric Excitation Supports Mammalian Retinal Motion Sensitivity

Graphical Abstract

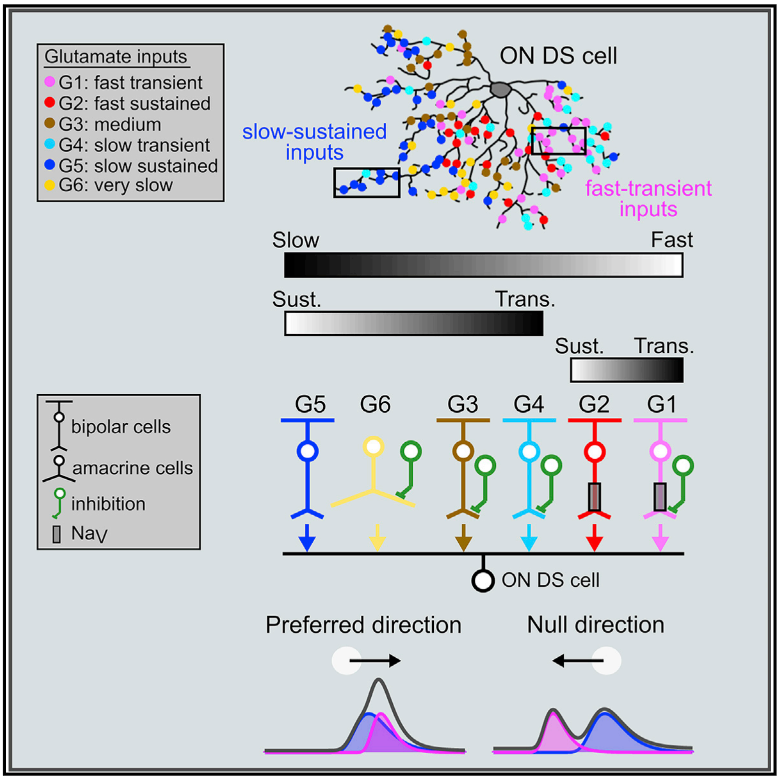

Highlights

- Space-time wiring between ON DS cells and bipolar cell types was identified
- Presynaptic inhibition and Na_V channels set the glutamate release dynamics
- Preferred direction enhancement supports the tuning to direction and slow speed
- Global dendritic summation is involved in computing image motion

Authors

Akihiro Matsumoto, Kevin L. Briggman, Keisuke Yonehara

Correspondence

keisuke.yonehara@dandrite.au.dk

In Brief

Matsumoto et al. find the space-time wiring between the dendrites of ON direction-selective cells and bipolar cell types in the mouse retina. Temporally diverse glutamatergic inputs, which are asymmetrically organized across the dendrites, are globally summated by a preferred direction enhancement mechanism for computing motion speed and direction.

Matsumoto et al., 2019, Current Biology *29*, 3277–3288
October 7, 2019 © 2019 The Author(s). Published by Elsevier Ltd.
https://doi.org/10.1016/j.cub.2019.08.048

Current Biology
Article

Spatiotemporally Asymmetric Excitation Supports Mammalian Retinal Motion Sensitivity

Akihiro Matsumoto,[1] Kevin L. Briggman,[2] and Keisuke Yonehara[1,3,*]
[1]Danish Research Institute of Translational Neuroscience–Nordic-EMBL Partnership for Molecular Medicine, Department of Biomedicine, Aarhus University, Ole Worms Allé 3, 8000 Aarhus C, Denmark
[2]Center of Advanced European Studies and Research (caesar), Ludwig-Erhard-Allee 2, 53175 Bonn, Germany
[3]Lead Contact
*Correspondence: keisuke.yonehara@dandrite.au.dk
https://doi.org/10.1016/j.cub.2019.08.048

SUMMARY

The detection of visual motion is a fundamental function of the visual system. How motion speed and direction are computed together at the cellular level, however, remains largely unknown. Here, we suggest a circuit mechanism by which excitatory inputs to direction-selective ganglion cells in the mouse retina become sensitive to the motion speed and direction of image motion. Electrophysiological, imaging, and connectomic analyses provide evidence that the dendrites of ON direction-selective cells receive spatially offset and asymmetrically filtered glutamatergic inputs along motion-preference axis from asymmetrically wired bipolar and amacrine cell types with distinct release dynamics. A computational model shows that, with this spatiotemporal structure, the input amplitude becomes sensitive to speed and direction by a preferred direction enhancement mechanism. Our results highlight the role of an excitatory mechanism in retinal motion computation by which feature selectivity emerges from non-selective inputs.

INTRODUCTION

The retina is the first stage in the mammalian nervous system in which visual motion is computed. Retinal direction-selective (DS) cells preferentially show spiking responses to visual stimuli moving in a particular direction (preferred direction) and show less spiking to the opposite, null direction [1]. It has been suggested that a key mechanism underlying retinal direction selectivity is null-direction suppression in DS cells implemented by spatially offset and DS GABAergic inhibitory inputs from starburst amacrine cells (SACs) [2–4]. In contrast to the well-described inhibitory inputs, the idea that excitatory inputs are also directionally selective remains controversial [5]. The DS cells receive glutamatergic and cholinergic excitatory inputs from bipolar and SACs, respectively [1, 6]. Studies with excitatory postsynaptic current (EPSC) recordings from DS cells have suggested directionally tuned glutamatergic inputs [7, 8]. However, a modeling study suggests that such apparent tunings could be an artifact due to imperfect voltage clamping [9]. Later studies with glutamate imaging from the inner plexiform layer or calcium imaging from bipolar cell axon terminals have indeed suggested that individual glutamatergic synaptic inputs are not directionally tuned [10–12], favoring the hypothesis of voltage clamping artifact (but see [13]).

DS cells in the optic lobe of the fly [14] or the visual cortical layer 4 of the mouse [15] use preferred-direction enhancement mechanisms in which untuned excitatory synaptic inputs are summated in a specific spatiotemporal manner to create tuned outputs as described by the Hassenstein-Reichardt model (Figure 1A) [14, 16]. The minimum requirement of the model is two presynaptic units with distinct delays converging to a postsynaptic cell. If the temporal difference by which two units separated by a distance (ΔS) are activated by a moving stimulus matches the difference in their delays (ΔT), the postsynaptic cell could effectively summate inputs, resulting in direction selectivity not in the time integral (Figure 1A, ii) but in the peak amplitude of input (Figure 1A, i). The Hassenstein-Reichardt model predicts a speed optimum; motion that is too slow or too fast should degrade the summation. A similar mechanism is also predicted to operate at connections between bipolar cells and starburst cell processes in the mouse retina: this may support the centrifugal direction selectivity of starburst cell processes [17–19] (but see [20]). However, spatiotemporal structure in the excitatory inputs to DS cells, which may support retinal motion sensitivity, remains to be explored.

Together with direction selectivity, speed selectivity is another fundamental visual feature represented by retinal DS cells [20–22]. Among the retinal DS cell types, ON DS cells that project their axons to the accessory optic system are adapted to detect slow global image motion induced by the self-movement of the animals: this mediates the optokinetic response, an eye or head movement reflex for gaze stabilization [23–26]. However, how these DS cells achieve their tuning to slow speed remains unknown. Here, we focus our study on glutamatergic inputs to the ON DS cells in the mouse retina to identify a mechanism involved in the extraction of motion speed and direction.

RESULTS

Diagonally Oriented Glutamatergic Space-Time Receptive Field

To examine how excitatory inputs may contribute to the speed and direction selectivity of ON DS cells, we performed two-photon targeted patch-clamp recordings from ON DS cells

This is an open access article under the CC BY license (http://creativecommons.org/licenses/by/4.0/).

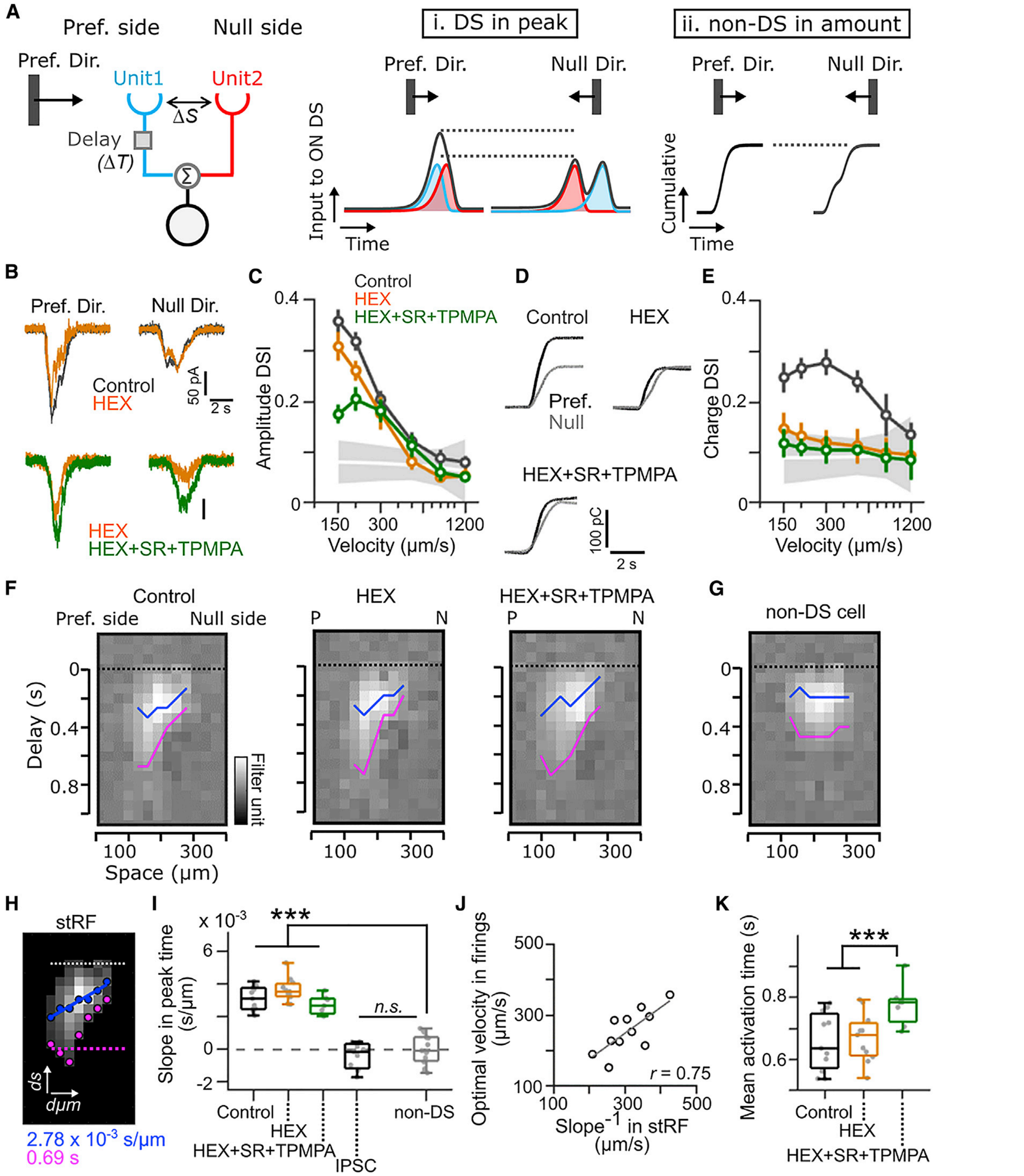

Figure 1. Receptive Field of Glutamatergic Input to ON DS Cells Is Oriented in Space Time

(A) Hassenstein-Reichardt model based on delayed input from unit 1 (blue) and input from unit 2 (red). The inputs to ON DS (black) are (i) DS in peak amplitude and (ii) non-DS in time integral of inputs.

(B) EPSCs recorded from ON DS cell during preferred and null direction motion at 200 μm/s. Gray, control; orange, with hexamethonium (HEX) (100 μM); green, with HEX, SR95531 (SR) (50 μM), and TPMPA (100 μM).

(C) Relationship between direction-selectivity index (DSI) in peak amplitude and motion speed. 16 ON DS cells are shown. Shaded gray, 95% confidence interval obtained from 16 non-DS cells. Error bars, SD.

(D) Cumulative time integral of EPSCs in control, HEX, and HEX+SR+TPMPA to preferred (black) and null direction (gray).

(E) Relationship between DSI in time integral (charge) and motion speed. Error bars, SD.

(F and G) Excitatory stRF (see Figures S1D–S1F) of an ON DS cell (F) and a non-DS cell (G). Peak time (blue) and activation time (magenta) are overlaid. Dotted line, synaptic input time.

(legend continued on next page)

genetically labeled in Hoxd10-GFP mice [27] (Figure S1A). We found that the peak amplitude of EPSCs at slow speed (<300 μm/s) had a higher direction selectivity than those recorded from randomly targeted non-DS cells (direction selective index [DSI], at 150 μm/s; 0.35 ± 0.08 in amplitude DSI; 0.27 ± 0.08 in charge DSI; $p < 0.001$; Mann-Whitney-Wilcoxon [MWW] test; Figures 1B–1E, black). The DS amplitude was maintained even after cholinergic receptors (hexamethonium [HEX]; 0.26 ± 0.05; $p = 2.087 \times 10^{-5}$; MWW test; orange) and $GABA_A$ and GABAc receptors (SR95531 [SR] and TPMPA; 0.21 ± 0.05; $p = 0.0025$; MWW test; green) were pharmacologically blocked, suggesting that the observed DS amplitude is not solely ascribed to voltage-clamping error related to DS inhibitory inputs (Figures S2A and S2B) [9]. Consistent with this idea, direction selectivity in the amplitude of inhibitory inputs showed weak speed tuning (Figures S2A–S2C). In contrast, the DS charge was lost at all velocities by blocking cholinergic receptors (Figures 1D and 1E, orange; see also Figures S2H and S2I; Discussion). These observations suggest that glutamatergic inputs are selective for speed and direction when the visual stimulus is moving slowly and support an idea that the selectivity is created by the preferred direction enhancement mechanism [14, 16] with linear summation. Indeed, blocking of cholinergic and GABA receptors did not abolish direction selectivity in the spiking responses at slow speed (<300 μm/s; Figures S2D–S2G).

Next, we mapped the spatiotemporal receptive field (stRF) for excitatory inputs using reverse correlation of dense noise stimuli [28] to explore potential asymmetry in the space-time input structure. The excitatory stRF in ON DS cell revealed a diagonally oriented profile of the stRF, being asymmetric along the motion preference axis (Figures 1F and 1G; peak time, blue line; activation time, magenta). The asymmetric time course was not sensitive to the cholinergic receptors blockade. To quantify the asymmetry in the space-time dimension, we measured slopes ($\Delta s/\Delta \mu m$) in stRF peak times (Figures 1H, 1I, and S1G). We found that the slopes of peak times in ON DS cells were significantly tilted compared with those in non-DS cells ($p < 0.001$; MWW test; Figures 1G and 1I), and the tilts were not lost by blocking cholinergic and GABAergic receptors ($p < 0.001$; Figure 1I, orange and green). The inhibitory stRF measured by inhibitory postsynaptic currents (IPSCs) to ON DS cells was not diagonally oriented (Figures 1I, black, and S2J), indicating that DS inhibition from SACs was not revealed by the dense noise stimulus. The optimal velocity predicted by the slope (slope^{-1} [$\Delta \mu m/\Delta s$]; Figure 1J) had a significant correlation with the optimal velocity in firings ($r = 0.76$; $p = 0.0092$; Figure S1H). Interestingly, the activation time became longer by blocking GABA receptors ($p < 0.001$; MWW test; Figure 1K, green), suggesting that GABAergic transmission is involved in filtering glutamatergic inputs. These results suggest that a spatiotemporally asymmetric structure of the glutamate-mediated receptive field may underlie the speed and direction selectivity of the EPSCs.

Functional Characterization of Glutamatergic Inputs

The identified spatiotemporal tilt of the stRF may suggest that EPSCs that arrived at the soma had been asymmetrically filtered along the motion-preference axis. This asymmetric filtering could be introduced by either presynaptic, synaptic, or dendritic mechanisms [5]. To test a potential role of presynaptic mechanisms, we monitored released glutamate using two-photon imaging [10, 12, 29]. We targeted the dendrites of genetically labeled ON DS cells in Pcdh9-Cre mice [30, 31] (Figure S1B) with the glutamate indicator iGluSnFr delivered by adeno-associated virus (AAV) (Figure 2). To estimate the temporal filtering property in the glutamate releases, we used a static flash stimulus that temporally modulates temporal frequency and contrast ("modulating flash") [29, 32]. To estimate the spatial RF profile, we used dense noise stimuli. The individual regions of interest (ROIs) detected along fluorescent-dye-filled dendrites (Figures 2A and S3) had diverse temporal and spatial RF shapes (Figure 2B).

To quantify the temporal dynamics of the glutamatergic signals, we clustered the ROIs into distinct groups by combining a sparse principal-component analysis (sPCA) and a Gaussian mixture model (see STAR Methods) [32]. We identified six ROI groups (G1–G6) with distinct temporal dynamics (Figures 2C–2E) and spatial and temporal RF properties (Figure 2F). The response decay (Figure 2G) and the sensitivity to temporal frequency and contrast (Figures 2H and 2I) of the six groups were heterogeneous. Next, we determined the distance between the six clusters based on a hierarchical clustering using the quantified features: peak latency; response decay; sensitivity to frequency and contrast; and temporal correlation between response and stimulus profile (Figure S3E). The dendrogram revealed G1 to be fast-transient, G2 fast-sustained, G3 medium, G4 slow-transient, and G5 slow-sustained types. G6 was prominently slow in peak latency, indicating that G6 may correspond to glutamatergic amacrine cells (GACs), which are known to provide inputs to ON DS cells [33, 34].

Presynaptic Inhibition to Establish Diversity of Temporal Dynamics in Glutamate Releases

The temporal dynamics of glutamatergic inputs could be shaped by intrinsic cellular mechanisms and/or inhibition of the axon terminals of presynaptic cells. Presynaptic inhibition may be mediated by small-field GABAergic and glycinergic amacrine cells and wide-field GABAergic amacrine cells that mediate surround suppression (Figure 3A) [29, 35–37]. To test the contribution of these inhibitory circuit motifs on the temporal dynamics of glutamate releases, we pharmacologically blocked GABA (Figures 3B–3D) and glycine receptors (Figures 3E–3H) with SR/TPMPA

(H) Thresholded stRF (see STAR Methods; Figure S1G). Peak time (blue dots) was fitted to calculate slopes. Activation time (magenta dots) was averaged to calculate mean activation time.

(I) Summary of slope in excitatory and inhibitory (IPSC) stRF of ON DS cells and non-DS cells. ***$p < 0.001$; n.s. > 0.05; Mann-Whitney-Wilcoxon test.

(J) Relationship between slope^{-1} in excitatory stRF and optimal velocity in firings.

(K) Summary of mean activation time. ***$p < 0.001$; Mann-Whitney-Wilcoxon test.

All averages are mean ± SD.

See also Figures S1 and S2.

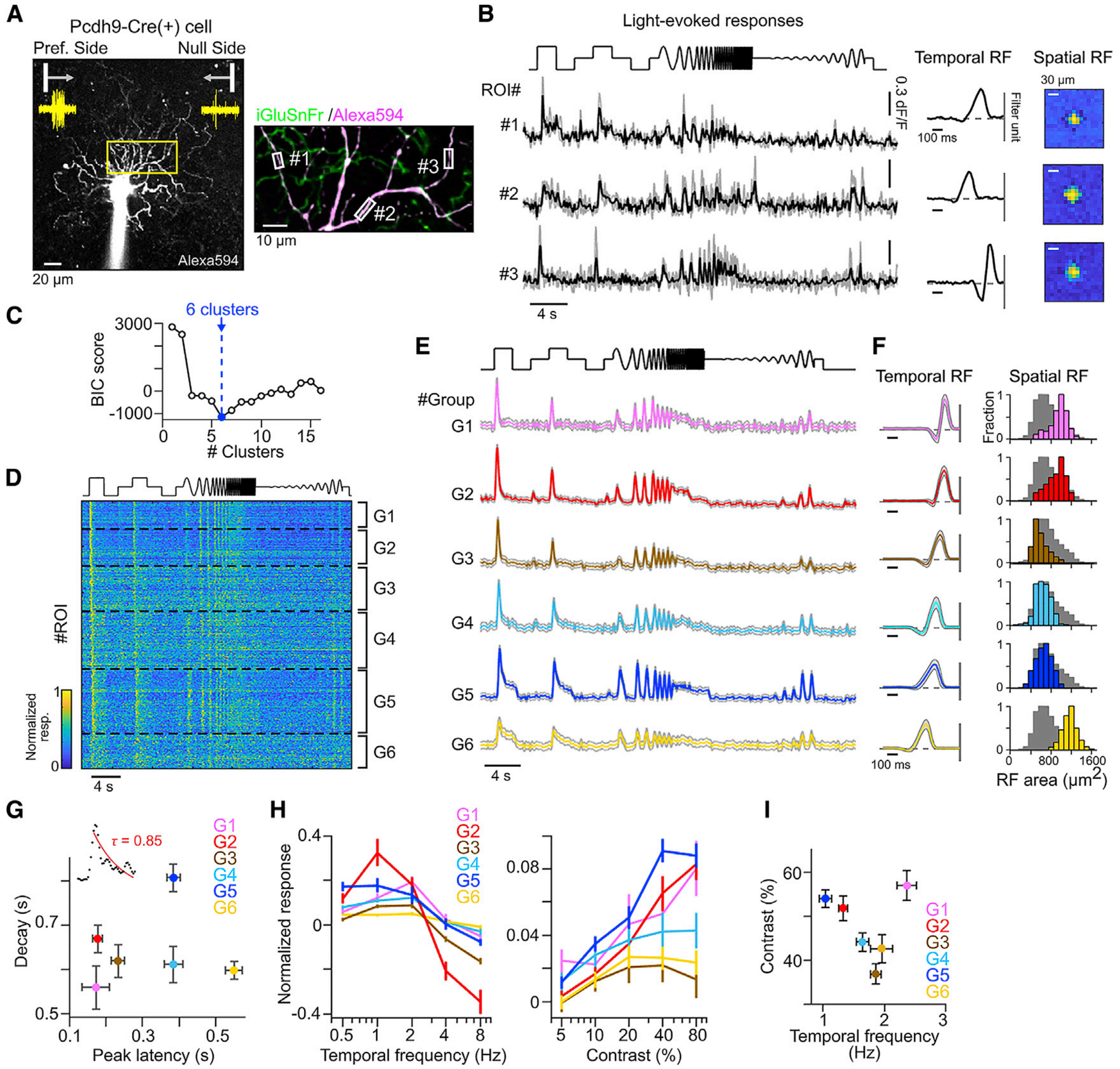

Figure 2. Functional Characterization of Glutamatergic Inputs to ON DS Cells

(A) Left: labeled ON DS cell with its firing responses to motion stimulus (yellow). Scale bar, 20 μm. Right: field of view (FOV rectangle in left). Rectangles, regions of interest (ROIs). Scale bar, 10 μm.

(B) Light-evoked glutamate signals (gray, each trial; black, averaged signal), temporal RF (gray line, event timing), and spatial RF (yellow) in example three ROIs shown in (A).

(C) Relationship between Bayesian information criterion score (see STAR Methods) and number of clusters in the Gaussian mixture model. Blue, estimated optimal number of clusters.

(D) A heatmap showing glutamate signals for the detected six clusters (G1–G6). Black dotted lines, borders of each cluster. 1,175 ROIs and 6 ON DS cells.

(E) Averaged light-evoked glutamate signals for the six clusters. Gray shade, SD.

(F) Temporal RF (left; gray shade, SD) and histograms of spatial RF area (right; gray shade, all ROIs).

(G) Decay and peak latency in responses to static flashing spot (diameter, 500 μm; 100% contrast). (Inset) Measured glutamate signal (black dots) and fitted exponential curve (red line) to calculate decay constant τ.

(H) Mean tunings to temporal frequency (left) and contrast (right) in the six clusters.

(I) Preferred contrast and frequency calculated by the tunings in individual ROIs.

Averages in (G)–(I) are mean ± SE.

See also Figure S3.

and strychnine, respectively, while imaging iGluSnFr using spot stimuli of different sizes (50–600 μm) and dense noise (Figure 3H). The sensitivity of G1–G6 to different conditions suggested the following: small-field GABAergic cells inhibit G1, G3, G4, and G6 terminals to shorten the decay (Figure 3C); small-field glycinergic cells inhibit G3 and G6 terminals (Figure 3F); and large-field GABAergic cells inhibit all groups for mediating surround suppression (Figures 3B and S4D). The suppression of G1 and G2 terminals was enhanced by the blocking of glycinergic inputs in response to the large (Figure 3E), but not the small, spot stimulation (Figure 3F), indicating that the large-field GABAergic cells inhibiting G1 and G2 terminals are inhibited by glycinergic cells

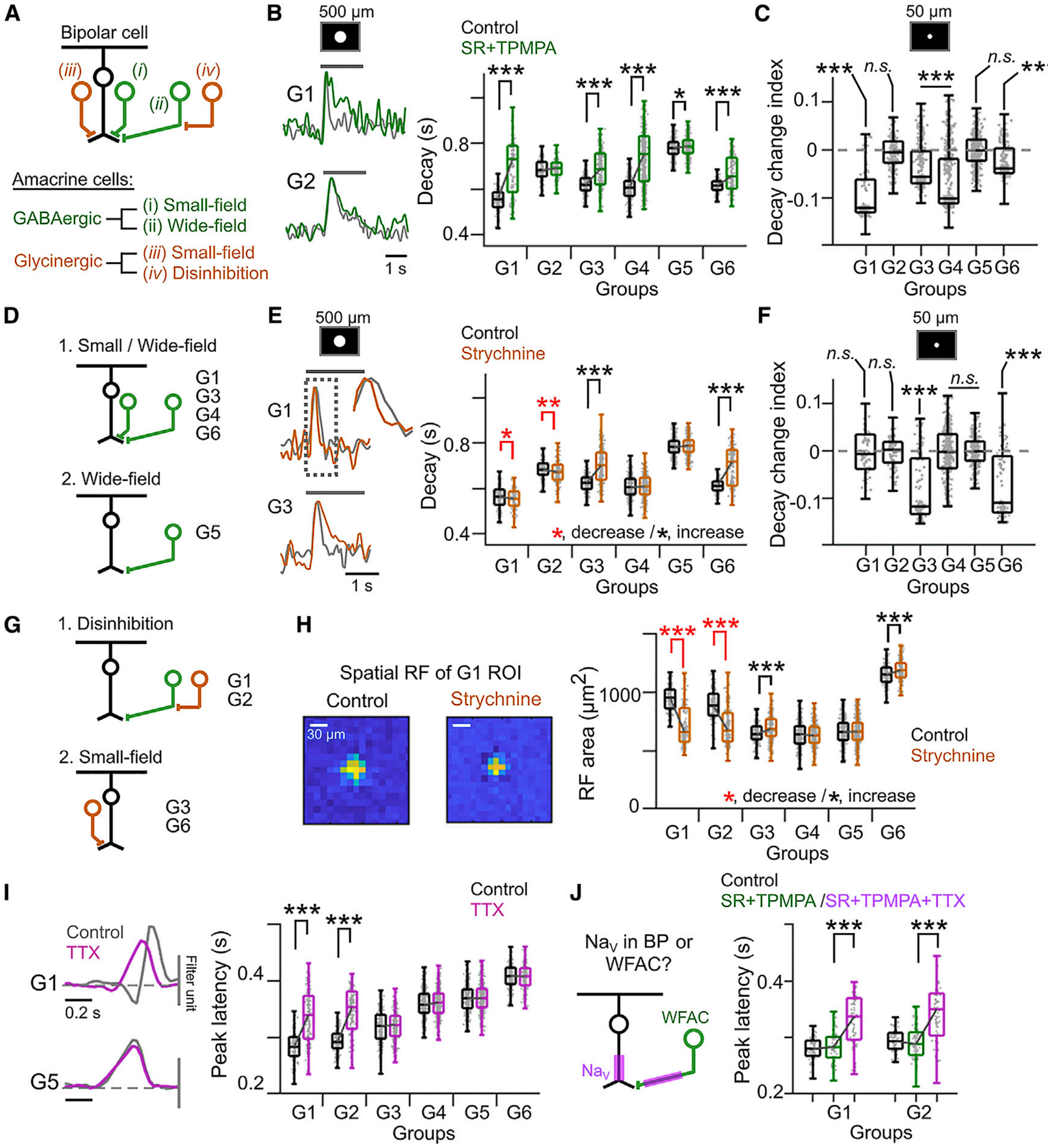

Figure 3. Pharmacological Dissection of Presynaptic Mechanisms

(A) Presynaptic inputs mediated by small-field (i) and wide-field (ii) GABAergic (green) and small-field glycinergic (iii; gold) amacrine cells. Inhibition of GABAergic cells by glycinergic cells (iv).

(B) Left: example glutamate signals (G1 and G2) during 500-μm flashing spot (gray line) in control (black) and SR+TPMPA (green). Amplitudes were normalized by the peaks. Right: summary of changes in response decay by GABA receptors blocking. Gray dots, individual ROIs. *p < 0.05; ***p < 0.001; One-tailed Wilcoxon signed-rank sum test.

(C) Decay change index (DCI) (see STAR Methods) to small spot (50 μm) with SR+TPMPA. Negative value in DCI indicates response decays are prolonged by GABA receptors blocking. ***p < 0.001; n.s. > 0.05; One-tailed Wilcoxon signed-rank sum test.

(D) Estimated GABAergic presynaptic inhibition.

(E) Left: example glutamate signals (G1 and G3) in control (black) and strychnine (1 μM; gold). Amplitudes were normalized by the peaks. Right: summary of the effects of glycine receptor blocking in response decay. *p < 0.05; **p < 0.01; ***p < 0.001; One-tailed Wilcoxon signed-rank sum test.

(F) DCI to small spot with strychnine. ***p < 0.001; n.s. > 0.05; One-tailed Wilcoxon signed-rank sum test.

(G) Estimated glycinergic presynaptic inhibition.

(H) Left: example spatial RF (G1) in control (top) and strychnine (bottom). Right: summary of changes in spatial RF area by glycine receptors blocking. Scale bars, 30 μm. ***p < 0.001; One-tailed Wilcoxon signed-rank sum test.

(legend continued on next page)

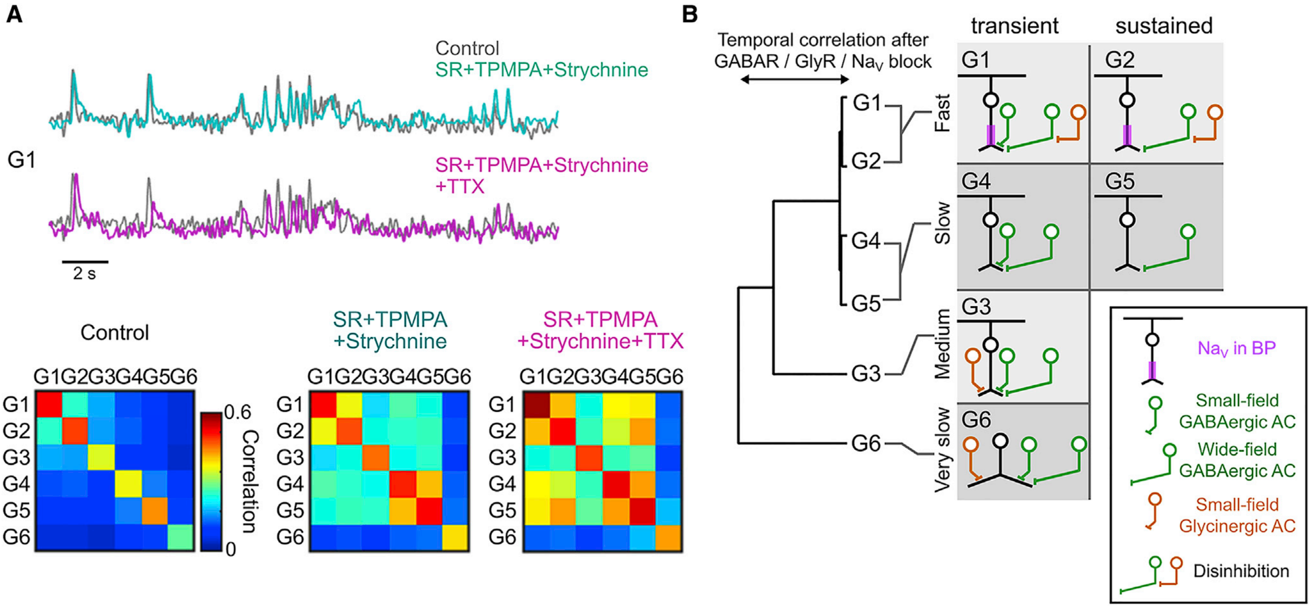

Figure 4. Presynaptic Mechanisms Shape Distinct Temporal Dynamics

(A) Top: example glutamate signal (G1) in control (gray), glycine, and GABA receptors blocked (cyan) and glycine, GABA receptors, and Na$_V$ blocked (purple). Bottom: mean temporal correlation in light-evoked glutamate signals among the six groups.

(B) Left: dendrogram estimated by a hierarchical clustering based on temporal correlation in light-evoked glutamate signals with blockers in (A). Right: schematic of presynaptic inputs and Na$_V$ expression.

See also Figure S4.

(Figure 3G). Indeed, the blocking of glycinergic inputs decreased the spatial RF size of G1 and G2 (Figure 3H) but increased that of G3 and G6 terminals, which rather receive direct glycinergic inputs.

In contrast to decay, peak latency was not affected by blocking inhibitory transmissions (Figure S4A). One possible mechanism for shaping peak latency would be the voltage-gated sodium channels (Na$_V$) expressed in specific bipolar cell types driving fast-action currents [38–41]. We found that the blocking of Na$_V$ by tetrodotoxin (TTX) significantly prolongs the latency in G1 and G2 (Figure 3I). These effects were not occluded by blocking GABA receptors in advance (Figure 3J, purple), indicating that the effects of Na$_V$ block were derived from bipolar cells mediating G1 and G2 rather than polyaxonal wide-field amacrine cells [42].

We investigated how presynaptic mechanisms and Na$_V$ contribute to shaping the dissimilar temporal dynamics of glutamate releases among ROI groups. Blocking both GABAergic and glycinergic transmissions resulted in better correlation between fast-transient G1 and fast-sustained G2 and between slow-transient G4 and slow-sustained G5 in response to a modulating flash stimulus (Figure 4A, cyan). Subsequent addition of TTX resulted in better correlation between fast G1 and G2 groups and slow G4 and G5 groups (Figure 4A, purple) and reduced discrepancies in the temporal filter among the six groups (Figures S4E–S4G). Hierarchical clustering of glutamate signals after blocking glycine, GABA receptors, and Na$_V$ channels (Figure 4B) support the idea that G1 and G2 form a Na$_V$-expressing fast subgroup and G4 and G5 form a slow subgroup. These results predict that the Na$_V$ blocking affects the summation of excitatory inputs. Indeed, the Na$_V$ blocking reduced the direction selectivity in EPSC (Figure S2K), reflecting the loss of latency differences between fast and slow groups in the excitatory stRF (Figures S2L and S2M). These results highlight the role of Na$_V$ in the fast subgroup in establishing temporal asymmetry.

Spatial Distribution of Glutamate Release on the Dendrites of ON DS Cell

To examine the spatial organization of the six detected ROI groups, we mapped their location on the dendrites of single ON DS cells visualized by dye applied with patch pipettes (Figure 5). Strikingly, the ROI groups were spatially biased along the motion-preference axis (Figures 5A–5C). Overall, the distribution had two layers of gradient from the preferred to null side: slow to fast and sustained to transient (Figures 5C and 5D). The slow-transient G4 and slow-sustained G5 are biased to the null and preferred side, respectively. The fast G1 and G2 groups are both biased to the null side, with the transient G1 group even more biased to the null side. The G3 group is biased

(I) Left: example temporal RFs (G1 and G5) in control (gray) and tetrodotoxin (TTX) (1 μM, purple). Right: summary of changes in peak latency in temporal RF by Na$_V$ blocking. ***$p < 0.001$; One-tailed Wilcoxon signed-rank sum test.

(J) Left: potential expression of Na$_V$ in bipolar cell (BP) or wide-field amacrine cell (WFAC). Right: peak latency in control (black), SR+TPMPA (green), and subsequent additional Na$_V$ blockade (purple). ***$p < 0.001$; One-tailed Wilcoxon signed-rank sum test.

See also Figure S4.

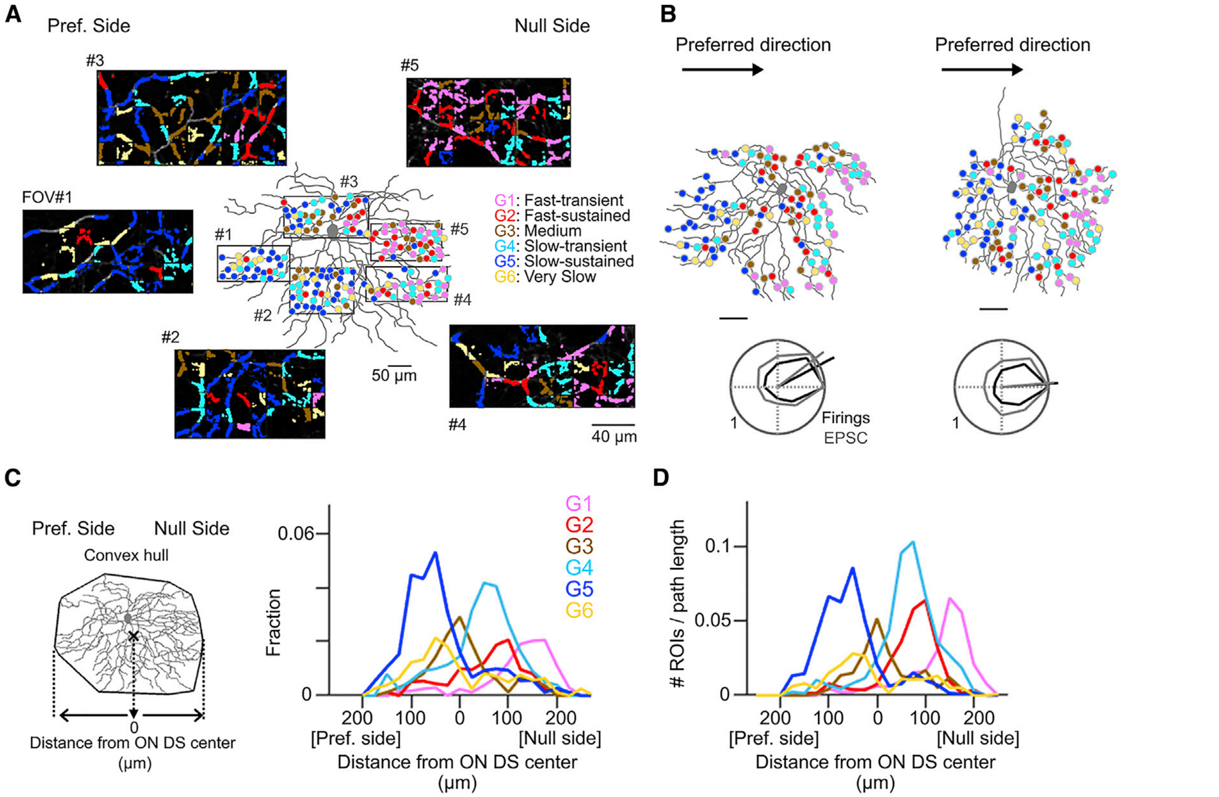

Figure 5. Spatiotemporally Organized Glutamatergic Inputs to ON DS Cells

(A) Distribution of glutamatergic inputs (colored circles) and five FOVs (rectangles) in an ON DS cell. Scale bar, 50 μm.

(B) Top: distribution of glutamatergic inputs in example two cells. Bottom: directional tunings of their firing activity (black) and EPSCs (gray). Scale bars, 50 μm.

(C) Left: center of dendritic field was determined from fitted convex hull. Right: histograms of ROI locations relative to the center of dendritic field. n = 6 cells.

(D) Histograms of ROI numbers normalized by dendritic path length.

See also Figures S5 and S6A–S6C.

to the center of the dendritic fields, and the G6 group showed a slight bias to the preferred side (Figures S6A–S6C).

Spatially Asymmetric Wiring between Bipolar Cell Types and ON DS Cells

To explore whether the functionally defined groups correspond to actual distinct bipolar cell types, we mapped the presynaptic connectivity of bipolar cells forming dyadic ribbon type synapses onto ON DS cells within a previously published serial block-face scanning electron microscopy (EM) volume [20]. The span of the volume (YY × ZZ μm^2) is smaller than a typical ON DS dendritic tree, so we focused on two partial dendritic reconstructions (Figure 6A) whose dendritic profiles were consistent with ON DS cells (Figure 6B). Mapping a sample of conventional synapses onto the ON DS cell trees revealed asymmetrically connected SACs that allowed us to infer the preferred null axis of the cells as previously described [3]. During this process, presynaptic neurons consistent with GACs were also identified [33, 34]. We then proceeded to map bipolar cell inputs (Figure 6C) and classified the cells by their axonal depth profiles and their placement in mosaics (Figure S5). We found inputs from bipolar cell types 5i, 5o, 5t, and 7. We then rotated the dendritic trees of the two ON DS fragments to align their inferred null direction axes and made an estimate of where the fragments would be positioned within a hypothetical 400-μm-diameter ON DS cell (Figure 6D). Finally, the synapse locations were projected onto the preferred null axis and we plotted the spatial histogram of the synapses along this axis (Figure 6E). The wiring pattern of the various sources of glutamatergic input showed a clear asymmetry along the preferred null axis. We observed the following correspondences between the functionally measured groups and the anatomically mapped cell types (compare Figure 5D versus 6E): G1 corresponded to bipolar cell type 5o, G2 to 5i, G3 to 7, and G6 to GAC inputs (Figure S6D). Bipolar cell type 5t synapses spanned the preferred and null side of the ON DS fragments and likely correspond to groups G4 and G5. Because G4 and G5 do not correspond to anatomically distinct bipolar cell classes, we hypothesize that differential presynaptic inhibition (Figure 4B) of bipolar cell type 5t synapses exists along the preferred null axis of ON DS cell.

Delay-and-Summate Model

To examine the causal relationship between the spatiotemporal organization of the glutamatergic inputs and the motion sensitivity of EPSCs in ON DS cells, we constructed a computational model based on a linear RF model [28] (Figure 7). The temporal filters estimated by glutamate imaging matched well with those estimated by EPSC (Figures S3F–S3H). The model described

CellPress

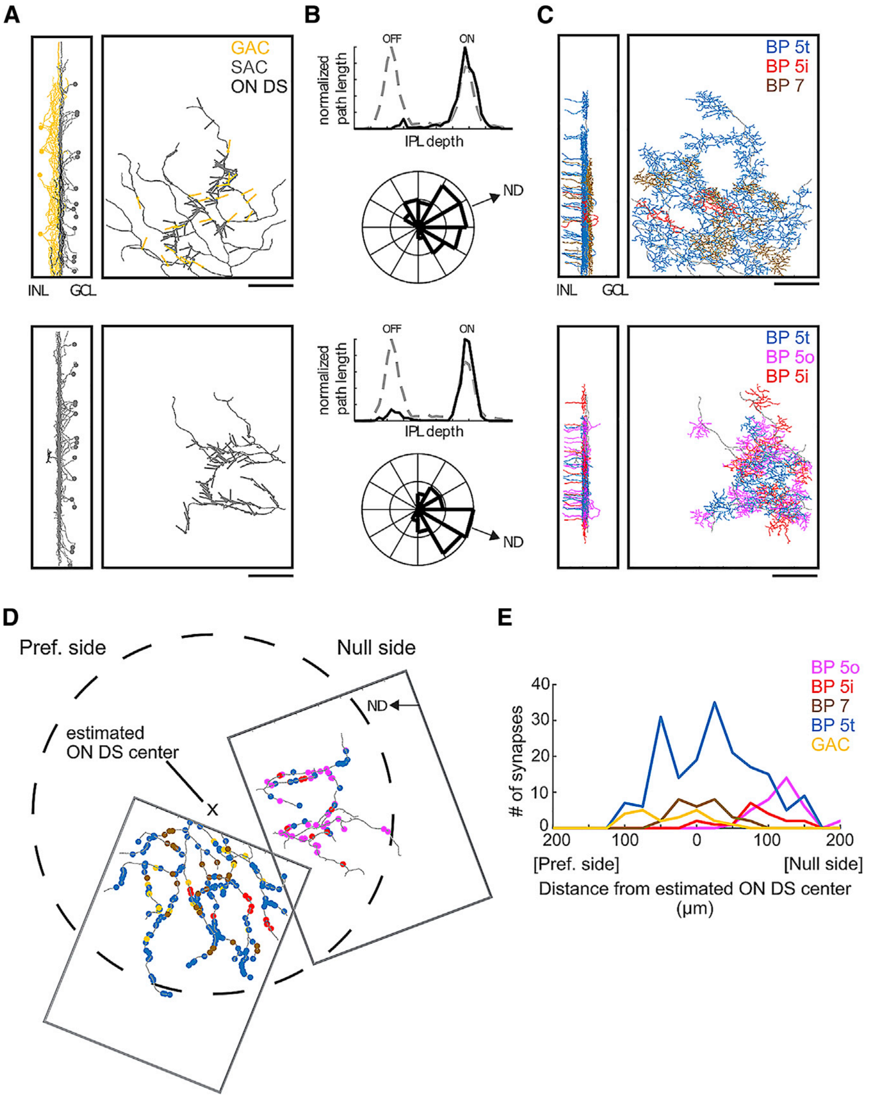

Figure 6. Anatomical Analysis of Excitatory Inputs to ON DS Cell

(A) Partial morphologies of two ON DS cells (black) contained within the EM volume. Left panel: a sample of presynaptic GACs (orange) and SACs (gray) that were presynaptic to the ON DS cell. Right panel: vectors indicating the directions of GAC (orange) and SAC (gray) presynaptic dendrites forming synapses (22 GAC synapses; 132 SAC synapses) onto the ON DS cell. Scale bars, 50 μm.

(B) Stratification profiles (top panels) of the ON DS cell (black) and an ON-OFF DS cell (gray dashed) from the same volume. Bottom panels: radial histograms of SAC dendrite angles and the inferred null directions (NDs).

(C) Bipolar cells (BPs) forming ribbon synapses onto the ON DS cell, color-coded by type 5t (blue), 5i (red), 5o (magenta), and 7 (brown). Scale bars, 50 μm.

(D) Locations of synapses formed by GACs (A; n = 22) and bipolar cells (C; n = 263), color-coded by type. EM volume rotated to align ND/PD axis horizontally and plotted over an estimate of the dendritic tree circumference (dashed line) of an ON DS cell with 400 μm diameter.

(E) Histogram of BP and GAC synapses projected onto the ND/PD axis of the ON DS cell (color-coded as in previous panels).

See also Figures S5 and S6D.

light-evoked signals in individual ROIs well (Figures 7A, S7A, and S7B). We created a delay-and-summate model ("delay-sum") based on the spatial distribution maps obtained by glutamate imaging. The simulated glutamate inputs were summed to represent gross excitatory inputs during motion stimulus ("EPSC"; Figures 7B and 7C). At a slow speed (200 μm/s), the model glutamate inputs were directionally selective in peak amplitude, but not in charge (Figure 7B). At a high speed (1,200 μm/s), neither the amplitude nor charge were directionally selective (Figure 7C). The model inputs peaked only after the moving stimulus passed the center of dendrites (black arrows in Figures 7B and 7C), indicating the global dendritic summation. Indeed, the firing probability during the preferred-direction motion peaked only when the moving stimulus entered the null side dendrites, although the firing responses started as soon as the moving stimulus entered the dendritic field (Figure 7D; see also Figures S7C–S7F; Discussion).

The model output to preferred-direction motion showed a clear slow-speed (200 μm/s) preference (Figure 7G, black), replicating EPSCs measured when cholinergic receptors were blocked (Figure 7G, purple band). The model DSI (Figure 7H, black) fitted well to the measured DSI in EPSCs (Figure 7H, purple band). On the other hand, a "shuffled" model, in which the location of ROIs was shuffled (Figure 7F), did not show any clear speed tuning (Figures 7G and 7H, gray). Furthermore, we created a "sustained" model, in which transient G1 and G4 were replaced with sustained G2 and G5, respectively (Figure 7F), to test the significance of spatially asymmetric small-field presynaptic GABAergic inhibition. In the sustained model, the speed tuning was slightly shifted to the higher speed (Figures 7G and 7H, green), replicating the result of blocking GABA receptors (Figure 7H, green band; see also Figure 1C). These results demonstrate the role of spatiotemporal organization of glutamatergic inputs in the computation of speed and direction and illuminate the role of spatially asymmetric presynaptic inhibition in establishing sharp speed tuning and robust direction selectivity.

DISCUSSION

These results suggest a circuit mechanism with which bipolar cells play a key role in retinal speed and direction selectivity. Our glutamate imaging identified two layers of spatially asymmetric filtering of glutamatergic inputs, namely fastness and slowness, and transience and sustainedness, along the motion-preference axis of ON DS cells. Our EM study demonstrated spatially asymmetric connectivity from four bipolar cell types and one GAC type to ON DS cells, providing a function-structure

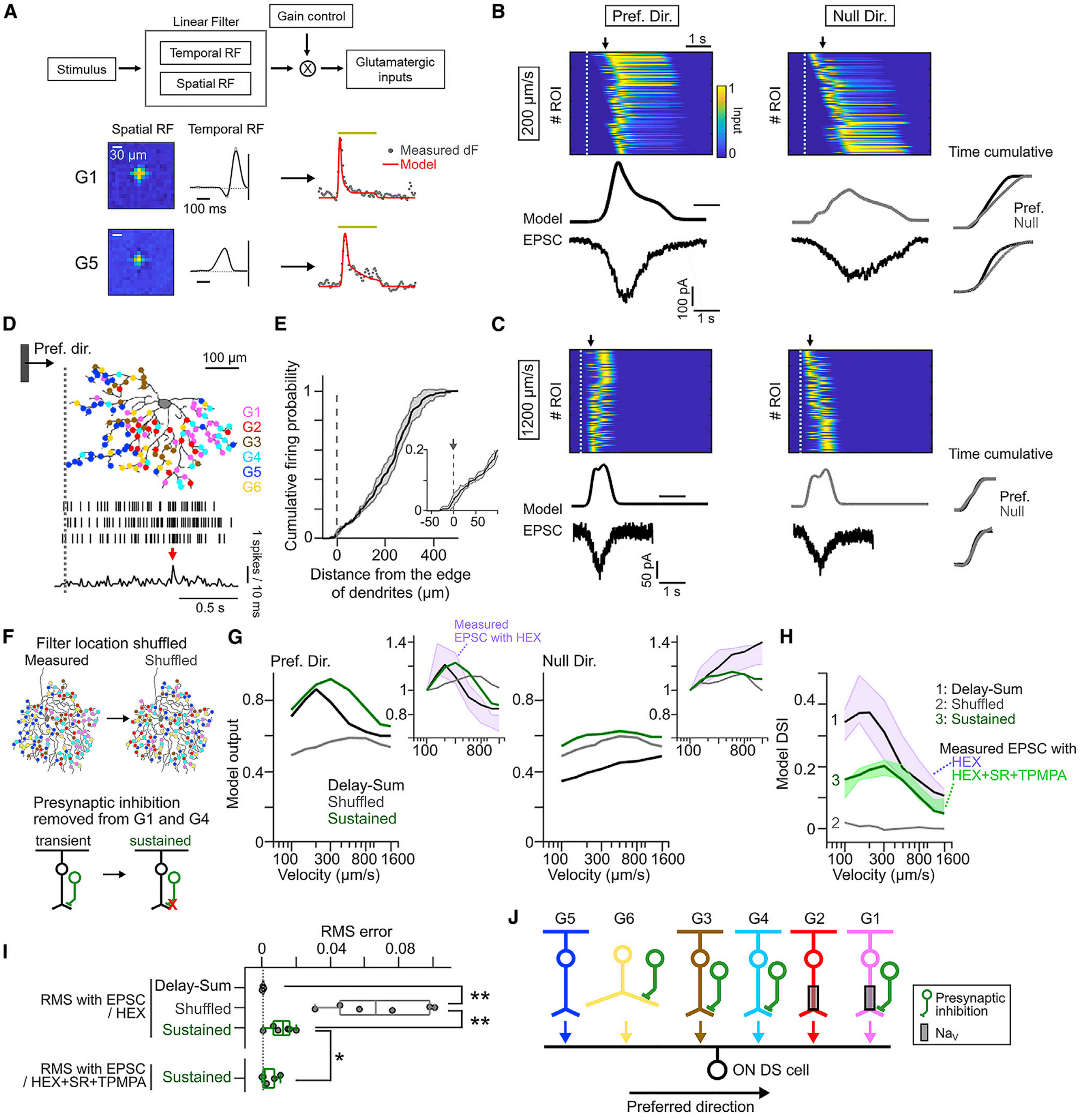

Figure 7. Delay-and-Summate Model for Speed- and Direction-Selective Excitations

(A) Top: modeling of glutamatergic input based on linear receptive field model (see STAR Methods). Bottom: example modeling in ROIs of G1 and G5 groups is shown. Using estimated spatial and temporal RF, measured (gray dots) glutamatergic inputs were simulated (red line). Scale bars, 30 µm.

(B and C) Simulated glutamatergic inputs to one ON DS cell during preferred (left) and null (right) directions at 200 µm/s (B) or 1,200 µm/s (C). Top, heatmaps show simulated individual glutamatergic inputs. Dotted white line, the timing when the leading edge of a moving stimulus enters a receptive field of glutamatergic unit at the most preferred (B) or null (C) side; black arrow, the timing when a moving stimulus passes a center of dendrites. Middle: a summated modeled input. Bottom: EPSC recorded from the ON DS cell during the corresponding motion stimulus. Right traces: time cumulative of modeled inputs (top) and EPSC (bottom) in preferred (black) and null (gray) directions. The model used spatial distribution of 32 G1, 25 G2, 26 G3, 34 G4, 56 G5, and 20 G6 ROIs measured from one ON DS cell.

(D) Relationship between firings (black lines in middle; 3 trials) and the location of leading edge of a moving stimulus in relation to the spatial distribution of glutamatergic inputs. Dotted line, the timing when the leading edge of a moving stimulus enters the dendritic field. Scale bar, 100 µm. Bottom: a peri-stimulus time histogram (10-ms bin width).

(legend continued on next page)

correlation. Our computer simulation indicates that ON DS cells become selective for motion speed and direction by a “delay-and-summate” mechanism, in which the dendrites summate spatiotemporally organized glutamatergic inputs with distinct fastness and slowness and transience and sustainedness. Note that the delay-and-summate mechanism explains the inputs to a postsynaptic cell, in contrast to the Hassenstein-Reichardt model, which generally explains the outputs of a postsynaptic cell. We cannot rule out synaptic or dendritic filtering mechanisms for introducing delays, but our work shows that diversity in the glutamate release dynamics of presynaptic cell types can sufficiently explain the summated EPSC dynamics. Furthermore, the speed and direction selectivity in EPSCs was well correlated with that in somatic spiking activity (Figures S2B and S2C).

The contribution of cholinergic inputs to the formation of asymmetric spatiotemporal receptive field and the amplitude DSI was not obvious (Figure 1). On the other hand, direction selectivity in the charge DSI was affected by the blocking of cholinergic receptors (Figure 1E). This could be explained by DS cholinergic releases from SAC [7]. Alternatively, cholinergic inputs in null-direction motion were shunted by the activated inhibitory conductance through GABAergic synapses due to imperfect voltage clamping of dendrites [3, 4, 9]. In fact, when the GABAergic receptors are blocked first, the additional blocking of cholinergic receptors did not affect the charge DSI of excitatory inputs (Figures S2H and S2I); this observation contradicts neither of these two possibilities. Future studies should investigate how the glutamatergic inputs interact with GABAergic and cholinergic inputs [6] at local dendrites to modulate the membrane potential of the dendrites and shape the output of the neurons in ON [43] and other DS cell types [44].

We showed that the spatiotemporal summation mechanism involves the integration of synaptic inputs across the global dendrites; the highest firing rates were achieved only by global summation (Figures 7B–7D). Nevertheless, it is still possible that the summation of inputs occurs in the local dendrites as well (Figures S6A and S6C). This idea was supported by the significant correlation between the EPSC and the modeled glutamatergic inputs in response to the local motion stimulus (Figures S7C–S7F). These results indicate that the ON DS cells utilize hierarchical summation mechanisms: local and global dendritic summations.

Glutamate imaging and EM reconstruction together suggest that fast-sustained G2, fast-transient G1, medium G3, and slow-sustained G5 and slow-transient G4 may correspond to type 5i, 5o, 7, and 5t bipolar cells, respectively (Figure S6D). This idea is further supported by our observation that response correlation between G4 and G5 becomes higher by pharmacologically blocking inhibitory circuits. The TTX sensitivity and tuning to high temporal frequency in G1 and G2 units (Figure 3) suggest that type 5i and 5o bipolar cells may correspond to previously reported type 5f bipolar cells [41].

If type 5t bipolar cells indeed correspond to G4 and G5, that would suggest an intriguing hypothesis that the axon terminal buttons of type 5t bipolar cells receive highly selective GABAergic presynaptic inhibition, where terminal boutons contacting the null-side dendrites of ON DS cells are selectively inhibited by small-field amacrine cells. In support of this idea, single bipolar cells are known to make synapses onto multiple subtypes of DS cells [45]. Because no synapses from SACs to the axons of bipolar cells have been identified [11, 20], it is likely that other types of GABAergic amacrine cells are involved in such terminal bouton-specific inhibition. Future work could explore the wiring rules of small-field and wide-field inhibitory amacrine cells presynaptic to these bipolar cell types.

How could ON DS cells achieve asymmetric connectivity with four bipolar cell types? For type 5i/o bipolar cell types, one simple solution would be that null-side dendrites of ON DS cells are tilted to catch these bipolar cell terminals, although we have not found such evidence. Alternatively, dendritic sector-specific synaptic adhesion molecules [46] could guide the precise wiring.

We conclude that motion computation by ON DS cells involves at least two circuit mechanisms: a preferred-direction enhancement mechanism, which is similar to the Hassenstein-Reichardt detector (delay-and-summate; Figure 7), implemented by bipolar cell types, and a null-direction suppression mechanism, which is similar to the Barlow-Levick detector, implemented by SACs. Why do the ON DS cells require both excitatory and inhibitory mechanisms for motion detection? First, it seems that the ON DS cells must accurately detect the velocity of slow retinal slip [47] to mediate the optokinetic reflex that works best at slow speed range. Importantly, the optimal speed of EPSCs predicted by our model (200 μm/s) matches well with the speeds at which the mouse optokinetic reflex shows optimal gain (<150 μm/s) [26]. These findings and another observation that inhibitory inputs are only moderately tuned to motion speed (Figure S2A) together support an idea that slow speed preference of ON DS cells largely depends on an excitatory delay-and-summate mechanism. Second, direction selectivity with utilizing only a glutamatergic mechanism may not be robust enough and has a limited speed range (Figure S2B). Third, the inhibitory mechanism likely ensures that entire dendritic segments can compute motion direction in response to local motion, regardless of the local geometry of bipolar inputs. A recent work demonstrated a role of glycinergic inhibition in speed tuning in rabbit ON DS

(E) Relationship between the distance from the edge of dendrites in preferred side and cumulative firing probability in preferred-direction motion. Black line and gray band, mean ± SD from 6 cells. Inset: firing probability around the edge of dendrites.

(F) Schematic of shuffled (top) and sustained models (bottom). In shuffled model, the spatial location of individual glutamatergic inputs was shuffled from measured distribution. In sustained model, transient G1 groups, G1 and G4, were replaced with sustained groups, G2 and G5, respectively.

(G) Speed tuning to preferred (left) and null direction (right) in delay-and-summate (black), shuffled (gray), and sustained (green) models. Inset: model inputs normalized by one at 100 μm/s. Purple band, mean ± SD of EPSC measured from ON DS cell.

(H) Speed tuning of model DSI. Purple and green bands, measured mean ± SD of DSI under HEX and HEX+SR+TPMPA conditions, respectively.

(I) Root mean square (RMS) error between measured DSI and model DSI. Gray circles, 6 cells. *$p < 0.05$; **$p < 0.01$; paired *t*-test.

(J) Wirings between glutamatergic cells (G1–G6) and ON DS cell.

See also Figures S6D and S7.

cells [48]. Therefore, it is likely that the speed tuning involves mechanisms based on a combination of glutamatergic excitation and feedforward glycinergic inhibition.

Strikingly, T4 and T5 cells in the optic lobe of a dipteran fly, which mediate optomotor responses [49], also use a combination of preferred-direction enhancement and null-direction suppression mechanisms for computing visual motion [5, 50, 51]. Therefore, our findings illuminate a fundamental computational solution employed from insects to mammals for detecting self-movement-induced visual motion.

STAR★METHODS

Detailed methods are provided in the online version of this paper and include the following:

- KEY RESOURCES TABLE
- LEAD CONTACT AND MATERIALS AVAILABILITY
- EXPERIMENTAL MODEL AND SUBJECT DETAILS
- METHOD DETAILS
  - Retinal preparation
  - Electrophysiology
  - Virus injections
  - Two-photon glutamate imaging
  - Visual stimulation
  - ROI detection
  - Receptive field estimation
  - Clustering
  - Connectomic reconstruction
  - Model simulation
- QUANTIFICATION AND STATISTICAL ANALYSIS
- DATA AND CODE AVAILABILITY

SUPPLEMENTAL INFORMATION

Supplemental Information can be found online at https://doi.org/10.1016/j.cub.2019.08.048.

ACKNOWLEDGMENTS

We thank B. Thomsen and M. Yonehara for technical assistance, E. Repak for building optical pathways for the visual stimulus, and Z. Raics for making the visual stimulus and helping with the maintenance of the imaging system. We thank Hiroki Asari, David Berson, Antonia Drinnenberg, Sara Oakeley, and Stuart Trenholm for commenting on the manuscript. This work was supported by Lundbeck Foundation (DANDRITE-R248-2016-2518), European Research Council Starting Grant (CIRCUITASSEMBLY no. 638730), Novo Nordisk Foundation (NNF15OC0017252), and Carlsberg Foundation (CF17-008) to K.Y.

AUTHOR CONTRIBUTIONS

A.M. and K.Y. designed the study. A.M. performed all retinal experiments and data analysis. K.Y. performed viral injections. K.L.B. performed the connectomic analysis. A.M., K.L.B., and K.Y. wrote the manuscript.

DECLARATION OF INTERESTS

The authors declare no competing interests.

Received: June 22, 2019
Revised: August 15, 2019
Accepted: August 20, 2019
Published: September 26, 2019

REFERENCES

1. Vaney, D.I., Sivyer, B., and Taylor, W.R. (2012). Direction selectivity in the retina: symmetry and asymmetry in structure and function. Nat. Rev. Neurosci. *13*, 194–208.
2. Euler, T., Detwiler, P.B., and Denk, W. (2002). Directionally selective calcium signals in dendrites of starburst amacrine cells. Nature *418*, 845–852.
3. Briggman, K.L., Helmstaedter, M., and Denk, W. (2011). Wiring specificity in the direction-selectivity circuit of the retina. Nature *471*, 183–188.
4. Yonehara, K., Balint, K., Noda, M., Nagel, G., Bamberg, E., and Roska, B. (2011). Spatially asymmetric reorganization of inhibition establishes a motion-sensitive circuit. Nature *469*, 407–410.
5. Mauss, A.S., Vlasits, A., Borst, A., and Feller, M. (2017). Visual circuits for direction selectivity. Annu. Rev. Neurosci. *40*, 211–230.
6. Sethuramanujam, S., McLaughlin, A.J., deRosenroll, G., Hoggarth, A., Schwab, D.J., and Awatramani, G.B. (2016). A central role for mixed acetylcholine/GABA transmission in direction coding in the retina. Neuron *90*, 1243–1256.
7. Fried, S.I., Münch, T.A., and Werblin, F.S. (2005). Directional selectivity is formed at multiple levels by laterally offset inhibition in the rabbit retina. Neuron *46*, 117–127.
8. Sun, W., Deng, Q., Levick, W.R., and He, S. (2006). ON direction-selective ganglion cells in the mouse retina. J. Physiol. *576*, 197–202.
9. Poleg-Polsky, A., and Diamond, J.S. (2011). Imperfect space clamp permits electrotonic interactions between inhibitory and excitatory synaptic conductances, distorting voltage clamp recordings. PLoS ONE *6*, e19463.
10. Yonehara, K., Farrow, K., Ghanem, A., Hillier, D., Balint, K., Teixeira, M., Jüttner, J., Noda, M., Neve, R.L., Conzelmann, K.-K., and Roska, B. (2013). The first stage of cardinal direction selectivity is localized to the dendrites of retinal ganglion cells. Neuron *79*, 1078–1085.
11. Chen, M., Lee, S., Park, S.J.H., Looger, L.L., and Zhou, Z.J. (2014). Receptive field properties of bipolar cell axon terminals in direction-selective sublaminas of the mouse retina. J. Neurophysiol. *112*, 1950–1962.
12. Park, S.J.H., Kim, I.-J., Looger, L.L., Demb, J.B., and Borghuis, B.G. (2014). Excitatory synaptic inputs to mouse on-off direction-selective retinal ganglion cells lack direction tuning. J. Neurosci. *34*, 3976–3981.
13. Percival, K.A., Venkataramani, S., Smith, R.G., and Taylor, W.R. (2019). Directional excitatory input to direction-selective ganglion cells in the rabbit retina. J. Comp. Neurol. *527*, 270–281.
14. Borst, A., and Helmstaedter, M. (2015). Common circuit design in fly and mammalian motion vision. Nat. Neurosci. *18*, 1067–1076.
15. Lien, A.D., and Scanziani, M. (2018). Cortical direction selectivity emerges at convergence of thalamic synapses. Nature *558*, 80–86.
16. Reichardt, W. (1987). Evaluation of optical motion information by movement detectors. J. Comp. Physiol. A Neuroethol. Sens. Neural Behav. Physiol. *161*, 533–547.
17. Kim, J.S., Greene, M.J., Zlateski, A., Lee, K., Richardson, M., Turaga, S.C., Purcaro, M., Balkam, M., Robinson, A., Behabadi, B.F., et al.; EyeWirers (2014). Space-time wiring specificity supports direction selectivity in the retina. Nature *509*, 331–336.
18. Greene, M.J., Kim, J.S., and Seung, H.S.; EyeWirers (2016). Analogous convergence of sustained and transient inputs in parallel on and off pathways for retinal motion computation. Cell Rep. *14*, 1892–1900.
19. Fransen, J.W., and Borghuis, B.G. (2017). Temporally diverse excitation generates direction-selective responses in ON- and OFF-type retinal starburst amacrine cells. Cell Rep. *18*, 1356–1365.
20. Ding, H., Smith, R.G., Poleg-Polsky, A., Diamond, J.S., and Briggman, K.L. (2016). Species-specific wiring for direction selectivity in the mammalian retina. Nature *535*, 105–110.
21. Barlow, H.B., Hill, R.M., and Levick, W.R. (1964). Retinal ganglion cells responding selectively to direction and speed of image motion in the rabbit. J. Physiol. *173*, 377–407.

22. Sivyer, B., van Wyk, M., Vaney, D.I., and Taylor, W.R. (2010). Synaptic inputs and timing underlying the velocity tuning of direction-selective ganglion cells in rabbit retina. J. Physiol. *588*, 3243–3253.

23. Oyster, C.W. (1968). The analysis of image motion by the rabbit retina. J. Physiol. *199*, 613–635.

24. Sun, L.O., Brady, C.M., Cahill, H., Al-Khindi, T., Sakuta, H., Dhande, O.S., Noda, M., Huberman, A.D., Nathans, J., and Kolodkin, A.L. (2015). Functional assembly of accessory optic system circuitry critical for compensatory eye movements. Neuron *86*, 971–984.

25. Yonehara, K., Fiscella, M., Drinnenberg, A., Esposti, F., Trenholm, S., Krol, J., Franke, F., Scherf, B.G., Kusnyerik, A., Müller, J., et al. (2016). Congenital nystagmus gene FRMD7 is necessary for establishing a neuronal circuit asymmetry for direction selectivity. Neuron *89*, 177–193.

26. Kretschmer, F., Tariq, M., Chatila, W., Wu, B., and Badea, T.C. (2017). Comparison of optomotor and optokinetic reflexes in mice. J. Neurophysiol. *118*, 300–316.

27. Dhande, O.S., Estevez, M.E., Quattrochi, L.E., El-Danaf, R.N., Nguyen, P.L., Berson, D.M., and Huberman, A.D. (2013). Genetic dissection of retinal inputs to brainstem nuclei controlling image stabilization. J. Neurosci. *33*, 17797–17813.

28. Baccus, S.A., Ölveczky, B.P., Manu, M., and Meister, M. (2008). A retinal circuit that computes object motion. J. Neurosci. *28*, 6807–6817.

29. Franke, K., Berens, P., Schubert, T., Bethge, M., Euler, T., and Baden, T. (2017). Inhibition decorrelates visual feature representations in the inner retina. Nature *542*, 439–444.

30. Martersteck, E.M., Hirokawa, K.E., Evarts, M., Bernard, A., Duan, X., Li, Y., Ng, L., Oh, S.W., Ouellette, B., Royall, J.J., et al. (2017). Diverse central projection patterns of retinal ganglion cells. Cell Rep. *18*, 2058–2072.

31. Lilley, B.N., Sabbah, S., Hunyara, J.L., Gribble, K.D., Al-Khindi, T., Xiong, J., Wu, Z., Berson, D.M., and Kolodkin, A.L. (2019). Genetic access to neurons in the accessory optic system reveals a role for Sema6A in midbrain circuitry mediating motion perception. J. Comp. Neurol. *527*, 282–296.

32. Baden, T., Berens, P., Franke, K., Román Rosón, M., Bethge, M., and Euler, T. (2016). The functional diversity of retinal ganglion cells in the mouse. Nature *529*, 345–350.

33. Lee, S., Chen, L., Chen, M., Ye, M., Seal, R.P., and Zhou, Z.J. (2014). An unconventional glutamatergic circuit in the retina formed by vGluT3 amacrine cells. Neuron *84*, 708–715.

34. Chen, M., Lee, S., and Zhou, Z.J. (2017). Local synaptic integration enables ON-OFF asymmetric and layer-specific visual information processing in vGluT3 amacrine cell dendrites. Proc. Natl. Acad. Sci. USA *114*, 11518–11523.

35. Lukasiewicz, P.D. (2005). Synaptic mechanisms that shape visual signaling at the inner retina. Prog. Brain Res. *147*, 205–218.

36. Werblin, F.S. (2011). The retinal hypercircuit: a repeating synaptic interactive motif underlying visual function. J. Physiol. *589*, 3691–3702.

37. Zhang, C., and McCall, M.A. (2012). Receptor targets of amacrine cells. Vis. Neurosci. *29*, 11–29.

38. Baden, T., Esposti, F., Nikolaev, A., and Lagnado, L. (2011). Spikes in retinal bipolar cells phase-lock to visual stimuli with millisecond precision. Curr. Biol. *21*, 1859–1869.

39. Baden, T., Berens, P., Bethge, M., and Euler, T. (2013). Spikes in mammalian bipolar cells support temporal layering of the inner retina. Curr. Biol. *23*, 48–52.

40. Saszik, S., and DeVries, S.H. (2012). A mammalian retinal bipolar cell uses both graded changes in membrane voltage and all-or-nothing Na+ spikes to encode light. J. Neurosci. *32*, 297–307.

41. Hellmer, C.B., Zhou, Y., Fyk-Kolodziej, B., Hu, Z., and Ichinose, T. (2016). Morphological and physiological analysis of type-5 and other bipolar cells in the mouse retina. Neuroscience *315*, 246–258.

42. Manookin, M.B., Puller, C., Rieke, F., Neitz, J., and Neitz, M. (2015). Distinctive receptive field and physiological properties of a wide-field amacrine cell in the macaque monkey retina. J. Neurophysiol. *114*, 1606–1616.

43. Sivyer, B., and Williams, S.R. (2013). Direction selectivity is computed by active dendritic integration in retinal ganglion cells. Nat. Neurosci. *16*, 1848–1856.

44. Trenholm, S., Johnson, K., Li, X., Smith, R.G., and Awatramani, G.B. (2011). Parallel mechanisms encode direction in the retina. Neuron *71*, 683–694.

45. Asari, H., and Meister, M. (2012). Divergence of visual channels in the inner retina. Nat. Neurosci. *15*, 1581–1589.

46. Duan, X., Krishnaswamy, A., Laboulaye, M.A., Liu, J., Peng, Y.-R., Yamagata, M., Toma, K., and Sanes, J.R. (2018). Cadherin combinations recruit dendrites of distinct retinal neurons to a shared interneuronal scaffold. Neuron *99*, 1145–1154.e6.

47. Iwashita, M., Kanai, R., Funabiki, K., Matsuda, K., and Hirano, T. (2001). Dynamic properties, interactions and adaptive modifications of vestibulo-ocular reflex and optokinetic response in mice. Neurosci. Res. *39*, 299–311.

48. Sivyer, B., Tomlinson, A., and Taylor, W.R. (2019). Simulated saccadic stimuli suppress ON-type direction-selective retinal ganglion cells via glycinergic inhibition. J. Neurosci. *39*, 4312–4322.

49. Bahl, A., Ammer, G., Schilling, T., and Borst, A. (2013). Object tracking in motion-blind flies. Nat. Neurosci. *16*, 730–738.

50. Haag, J., Arenz, A., Serbe, E., Gabbiani, F., and Borst, A. (2016). Complementary mechanisms create direction selectivity in the fly. eLife *5*, e17421.

51. Leong, J.C.S., Esch, J.J., Poole, B., Ganguli, S., and Clandinin, T.R. (2016). Direction selectivity in Drosophila emerges from preferred-direction enhancement and null-direction suppression. J. Neurosci. *36*, 8078–8092.

52. Farrow, K., Teixeira, M., Szikra, T., Viney, T.J., Balint, K., Yonehara, K., and Roska, B. (2013). Ambient illumination toggles a neuronal circuit switch in the retina and visual perception at cone threshold. Neuron *78*, 325–338.

53. Zou, H., Hastie, T., and Tibshirani, R. (2006). Sparse principal component analysis. J. Comput. Graph. Stat. *15*, 265–286.

54. Fraley, C., and Raftery, A.E. (2002). Model-based clustering, discriminant analysis, and density estimation. J. Am. Stat. *97*, 611–631.

55. Helmstaedter, M., Briggman, K.L., and Denk, W. (2011). High-accuracy neurite reconstruction for high-throughput neuroanatomy. Nat. Neurosci. *14*, 1081–1088.

STAR★METHODS

KEY RESOURCES TABLE

REAGENT or RESOURCE	SOURCE	IDENTIFIER
Bacterial and Virus Strains		
AAV9.hSyn.Flex.iGluSnFr.WPRE.SV40	Penn Vector Core	Cat# 98931-AAV9
Chemicals, Peptides, and Recombinant Proteins		
Fentanyl	Hameln	Cat# 007007
Midazolam	Hameln	Cat# 002085
Medetomidine	Hameln	Cat# 087896
Flumazenil	Hameln	Cat# 036259
Atipamezole	Orion Pharma	Cat# 471953
BAPTA	Sigma	Cat# A4926
QX-314-Br	Sigma	Cat# L5783
Neurobiotin	Vector Laboratories	Cat# SP-1120
Alexa 594	ThermoFisher	Cat# A10438
SR95531	Sigma	Cat# S106
TPMPA	Sigma	Cat# T200
Strychnine	Sigma	Cat# S0532
Hexamethonium bromide	Sigma	Cat# H0879
Tetrodotoxin	Tocris	Cat# 1078
Experimental Models: Organisms/Strains		
Mouse: C57BL/6J	Janvier Labs	C57BL/6JRj
Mouse: Hoxd10-EGFP	Mutant Mouse Research and Resource Centers	RRID: MMRRC_032065-UCD
Mouse: Pcdh9-Cre	Mutant Mouse Research and Resource Centers	RRID: MMRRC_036084-UCD
Software and Algorithms		
MATLAB 2017b	MathWorks	https://se.mathworks.com/products/matlab
LabVIEW	National Instruments	http://www.ni.com/labview
Python	Python Software Foundation	https://www.python.org/
Visual Stimulation	SELS Software (by Zoltan Raics)	http://raics.hu/zoltan/doku.php?id=software
Imaging	SELS Software (by Zoltan Raics)	http://raics.hu/zoltan/doku.php?id=software
SpaSM toolbox	Open Source	http://www2.imm.dtu.dk/projects/spasm/
LinLab 2	Scientifica	https://www.scientifica.uk.com/products/scientifica-linlab-2
MC700B Commander	Molecular Devices	http://mdc.custhelp.com/app/answers/detail/a_id/20059/related/1/session/L2F2LzEvdGltZS8xNTU1NzU0NzI0L3NpZC9OKjhXTEljbw%3D%3D
SPOT imaging software	SPOT Imaging Solutions	http://207.58.136.70/resources/downloads/index.php
Knossos software package	Open Source	https://knossostool.org/
Other		
Borosilicate glass micropipettes	Sutter Instruments	Item# BF100-50-10
Picospritzer III	Parker	Cat# 051-0530-900

LEAD CONTACT AND MATERIALS AVAILABILITY

Further information and requests for resources and reagents should be de directed to and will be fulfilled by the Lead Contact, Keisuke Yonehara (keisuke.yonehara@dandrite.au.dk). This study did not generate new unique reagents.

EXPERIMENTAL MODEL AND SUBJECT DETAILS

Wild-type mice (C57BL/6J) were obtained from Janvier labs. Hoxd10-EGFP [27] and Pcdh9-Cre [30, 31] mice were obtained from Mutant Mouse Research and Resource Centers (strains: STOCK Tg(Hoxd10-EGFP)LT174Gsat/Mmucd and STOCK Tg(Pcdh-9-cre)NP276Gsat/Mmucd) and backcrossed to C57BL/6J mice for more than 5 generations. We used 4- to 16-week-old mice of either sex. Mice were group housed throughout and maintained in a 12-hour/12-hour light/dark cycle with *ad libitum* access to food and water. All animal experiments were performed according to standard ethical guidelines and were approved by the Danish National Animal Experiment Committee (Permission No. 2015−15−0201−00541).

METHOD DETAILS

Retinal preparation

Retinas were isolated from the left eye of mice dark-adapted for 1 hour before experiments. The isolated retina was mounted on a small piece of filter paper (MF-membrane, Millipore), in which a 2 × 2 mm window had been cut, with the ganglion cell side up. During the procedure, the retina was illuminated by dim red light (KL 1600 LED, Olympus) filtered with a 650 ± 45 nm band-pass optical filter (ET650/45 × ,Chroma) and bathed in Ringer's medium (in mM): 110 NaCl, 2.5 KCl, 1 $CaCl_2$, 1.6 $MgCl_2$, 10 D-glucose, 22 $NaHCO_3$ bubbled with 5% CO_2, 95% O_2. The retina was kept at 35-36°C and continuously superfused with oxygenated Ringer's medium during recordings.

Electrophysiology

Electrophysiological recordings were conducted with an Axon Multiclamp 700 B amplifier (Molecular Devices). Signals were acquired using customized software on LabVIEW (National Instruments) developed by Zoltan Raics (SELS Software), and digitized at 10 kHz. Borosilicate glass micropipettes pulled by a micropipette puller (P-97, Sutter Instrument) were used for recordings. The firing discharges were recorded in cell-attached mode using pipettes filled with the Ringer's medium, and synaptic currents were recorded in whole-cell clamp mode filled with intracellular solution (in mM): 112.5 $CsCH_3SO_3$, 1 $MgSO_4$, 7.8 × 10^{-3} $CaCl_2$, 0.5 BAPTA, 10 HEPES, 4 ATP-Na_2, 0.5 GTP-Na_3, 5 QX314-Br, 7.5 neurobiotin chloride. pH was adjusted to 7.2 with CsOH. The equilibrium potential for chloride was calculated to be ∼−60 mV. Membrane potentials were held at −60 mV for recording the excitatory postsynaptic current (EPSC) and 0 mV for recording the inhibitory postsynaptic current (IPSC). The resistance of pipettes was 3-5 and 6-10 mOhm for cell-attached and whole-cell recording, respectively. To visualize the dendrites of recorded neurons, Alexa 594 (10 μM, ThermoFisher) was added to the intracellular solution. The labeled GFP cells were targeted for recordings using a two-photon microscope equipped with a mode-locked Ti:sapphire laser (Mai Tai DeepSee, Spectra Physics), set to 940 nm, integrated into the physiological recording setup (SliceScope, Scientifica), as described previously [10, 52]. The two-photon fluorescence image was overlaid on the infra-red (IR) image acquired by a CCD camera (RT3, SPOT Imaging). The IR light was generated by a digital light projector (NP-V311X, NEC) with a 750 ± 25 nm filter.

For pharmacological experiments, we used SR95531 (50 μM, Sigma) to bock $GABA_A$ receptors, TPMPA (100 μM, Sigma) to block $GABA_C$ receptors, strychnine (1 μM, Sigma) to block glycine receptors, hexamethonium bromide (2 μM, Sigma) to block nicotinic acetylcholine receptors, and tetrodotoxin (1 μM, Tocris) to block Na^+ channels. These agents were bath-applied during recordings.

Virus injections

AAV9.hSyn.Flex.iGluSnFr.WPRE.SV40 (7.73 × 10^{13} GC/ml) was obtained from Penn Vector Core (#98931). For intravitreal viral injections mice, were anesthetized with an i.p. injection of fentanyl (0.05 mg/kg body weight; Actavi), midazolam (5.0 mg/kg body weight; Dormicum, Roche) and medetomidine (0.5 mg/kg body weight; Domitor, Orion) mixture dissolved in saline. We made a small hole at the border between the sclera and the cornea with a 30-gauge needle. Next, we loaded the AAV into a pulled borosilicate glass micropipette (30 μm tip diameter), and 2 μl was pressure-injected through the hole into the vitreous of the left eye using a Picospritzer III (Parker). Mice were returned to their home cage after anesthesia was antagonized by an i.p. injection of a flumazenil (0.5 mg/kg body weight; Anexate, Roche) and atipamezole (2.5 mg/kg body weight; Antisedan, Orion Pharma) mixture dissolved in saline and, after recovering, were placed on a heating pad for one hour.

Two-photon glutamate imaging

Three to four weeks after virus injection, we performed two-photon glutamate imaging. The isolated retina was placed under the microscope (SliceScope, Scientifica) equipped with a galvo-galvo scanning mirror system, a mode-locked Ti:Sapphire laser tuned to 940 nm (MaiTai DeepSee, Spectra-Physics), and an Olympus 20 × (1.0 NA) objective. The retina was superfused with oxygenated Ringer's medium. The iGluSnFr signals emitted were passed through a set of optical filters (ET525/50 m, Chroma; lp GG495, Schott) and collected with a GaAsP detector. Images were acquired at 8-15 Hz using custom software developed by Zoltan Raics (SELS Software). Temporal information about scan timings was recorded by TTL signals generated at the end of each scan, and the scan timing and visual stimulus timing were subsequently aligned during offline analysis.

Visual stimulation

The visual stimulation was generated via custom-made software (Python and LabVIEW) developed by Zoltan Raics. For electrophysiological recordings, the stimulus was projected through a DLP projector (NP-V311X, NEC). The stimulus was focused onto the photoreceptor layer of the mounted retina through a condenser (WI-DICD, Olympus). The intensity was measured using a photodiode power meter (Thorlabs), and the power of the spectrum was measured using a spectrometer (Ocean Optics). The calculated photoisomerization rate ranged from 0.0025 to 0.01 × 10^7 photons absorbed per rod per second (R*/s) both for electrophysiological recordings and two-photon imaging. For glutamate imaging, the stimulus was projected using a DLP projector (LightCrafter Fiber E4500 MKII, EKB Technologies) coupled via a liquid light guide to an LED source (4-Wavelength High-Power LED Source, Thorlabs) with a 400 nm LED (LZ4-00UA00, LED Engin) through a band-pass optical filter (ET405/40 × ,Chroma). The stimuli were exclusively presented during the fly-back period of the horizontal scanning mirror [52]. The contrast of visual stimulus ($C_{stimulus}$) was calculated as,

$$C_{stimulus} = \left(L_{stimulus} - L_{background}\right) / \left(L_{stimulus} + L_{background}\right)$$

in which L indicates intensity.

We used four light stimulus patterns: static spot (50-600 μm in diameter, 2 s in duration, 100% positive contrast, Figure 3), modulating flash (500 μm, Figures 2 and 3) [29, 32], dense noise (Figures 1, 2, and 3), and moving spot (300 μm in diameter, 100% positive contrast) in eight directions (0-315°, Δ45°) at 150-1200 μm/s (Figure 1). The modulating flash had four phases: static flashing spot of 100% contrast, one of 50% contrast, one with increasing temporal frequency from 0.5 to 8 Hz, and one with increasing contrast from 5 to 80%. The dense noise was constructed from black and white pixels (for glutamate imaging, 10-20 μm in length; for electrophysiological recording, 30-50 μm, 20 × 20 matrix), each flickering randomly (for glutamate imaging, 10-20 Hz; for electrophysiological recording, 20-30 Hz). For the local motion (Figure S7), we set three stimulation windows, each of which was 150 μm in width × 400 μm in length, at the preferred side, center, and null side of the ON DS cell dendrites (Figure S7A).

Using the static spot and modulating flash, we calculated five parameters as response properties (Figures 2 and 3): peak latency and decay in response to static flash of 100% contrast; frequency and contrast sensitivity in response to frequency and contrast modulating flash; and temporal correlation between response and stimulus profile. The response decay was obtained by fitting an exponential function to the measured glutamate signal (Figure 2G). To quantify changes of decay by pharmacological blockade (Figures 3C, 3F, and S4A–S4D), we used a decay change index (DCI):

$$DCI = \left(\tau_{control} - \tau_{blocker}\right) / \left(\tau_{control} + \tau_{blockre}\right)$$

where $\tau_{control}$ and $\tau_{blocker}$ were the calculated decays under the control and blocker conditions, respectively. To evaluate the changes of temporal filter properties, we used peak latency, peak amplitude, and input amount to calculate the same index (Change index in Figures S4A–S4C). To quantify the frequency and contrast sensitivity in response to a modulating flash, we calculated the mean response strength before the start of each phase (1 s) as the baseline strength, and the peak response amplitude during the modulating phases was divided by the baseline strength. To quantify the preferred frequency and contrast (Figure 2I), the tuning curve of each ROI was fitted by a polynominal curve based on the least-square method in MATLAB. Based on the fitted curve, the peaks in frequency and contrast were defined as preferred frequency and contrast, respectively.

To measure directional tuning and motion speed preference, we used a spot (300 μm in diameter, 100% positive contrast) moving in eight directions (0-315°, Δ45°) at 150-1200 μm/s. To quantify the directional selectivity, we used a direction selectivity index (DSI):

$$DSI = \left(Resp_{pref} - Resp_{null}\right) / \left(Resp_{pref} + Resp_{null}\right)$$

in which $Resp_X$ is the maximum response during motion direction X. The firing DSIs of ON DS cells were more than 0.3 (150 μm/s, Figure S1C). We defined the cells whose firing DSIs were lower than 0.2 in response to a spot moving at 150-1200 μm/s as non-direction selective (non-DS) cells. A preferred direction was defined as a direction which evoked maximum responses, and the opposite direction as the null direction.

ROI detection

Regions of interests (ROIs) for glutamate signals were determined by customized programs in MATLAB. First, the stack of acquired images was filtered with a Gaussian filter (3 × 3 pixels), and then each image was downsampled to 0.7 of the original using a MATLAB downsample function. After the calculation, responsive pixels were detected based on a threshold, mean + 3 SD. The glutamate signals for each responsive pixel detected were resampled using the MATLAB interp function. We calculated the temporal correlation among the resampled glutamate signals in pixels, and plotted the correlation coefficient against the distance between pixels. We set a threshold of correlation strength to determine which pixels were to be included as a single ROI. As in previous studies, we restricted the size of ROIs to a range between 1 and 10 μm^2 to match the size with that of bipolar cell axonal terminal boutons [29] (Figure S3B). The trace of raw glutamate signals was measured as mean fluorescence changes of pixels within an ROI. The signal was divided by the spontaneous trace before visual stimulation to calculate the glutamate signal used in analysis.

Receptive field estimation

We used a reverse correlation method based on dense noise stimulus [28, 29, 32]. To estimate the receptive field for postsynaptic currents to retinal ganglion cells, we calculated the weighted average of the synaptic inputs [28],

$$F(x, y, \tau) = \int^{\tau} r(t) S(x, y, t + \tau) d\tau$$

where $F(x,y,\tau)$ is the receptive field at location (x,y) at delay τ, $r(t)$ is postsynaptic currents, and $S(x,y,t)$ is the stimulus input at location (x,y).

To estimate the spatiotemporal receptive fields (stRFs) for postsynaptic inputs (Figure 1), we first determined the motion-preference axis (preferred to null side) from firing activity in response to a moving spot. We determined the receptive field center as a pixel with the highest value, and then we obtained the spatial profile as a rectangular area (30-50 μm in width × 600-1000 in length; Figure S1D, red square) of the spatial receptive field along the motion-preference axis. The temporal changes of the spatial profile revealed the stRF. To determine the stRF of non-DS cells (Figure 1G), those with DSI < 0.1, we determined the direction which evoked the maximum firing number and the opposite direction, and obtained the temporal changes of the spatial region in the same way as for DS cells. To determine the stRF slope (Figures 1H and S1G), we obtained signal pixels based on a threshold for each cell of mean + 3 SD of uncorrelated intensity, which was calculated by the reverse correlation between the event timing and newly generated independent dense noise (Figures S1E and S1F). We obtained a slope (Δs / Δμm) by linear fitting to the detected signal pixels using the least-square method (Figures 1H and S1G).

To estimate the receptive field for glutamate signals, we detected glutamate transient events based on a threshold (mean + 3 SD), and calculated event-triggered averages as spatial receptive fields. The edges of the estimated spatial receptive field were detected using the image processing toolbox in MATLAB, and fitted by a 2D Gaussian using the least-square method. To determine the size of the spatial receptive field (Figures 2 and 3), we thresholded the spatial receptive field in the same way as the stRF for synaptic inputs, and calculated the area of the signal pixels detected. To estimate the temporal receptive field, we calculated the average of 3 × 3 pixels neighboring a receptive field center pixel which had the highest intensity, and then we calculated temporal changes in intensity.

To compare the temporal filters estimated by postsynaptic currents (Figure 1) and glutamate signal (Figure 2), temporal filters in the stRF were separated into three parts: preferred side, center, and null side (Figures S3F and S3G). We first detected the three pixel in the thresholded stRF: a center pixel showing the maximum filter unit and pixels in the most preferred and null side with significant filter unit. The center parts contained three columns in the stRF: one column including the center pixel, and two columns displaced from the center column to either the prederred or null side. The preferred or null side columns spanned each from the center to the most preferred or null side, respectively. The temporal filter was an average of temporal filters within each three parts (Figure S3G). The temporal filters in glutamate signals were separated and averaged among fast groups in the null side (G1 and G2), medium groups in the center (G3), and slow groups in the preferred side (G4, G5) (Figure S3H).

To characterize the shape of a temporal receptive field in glutamate imaging, we calculated time-to-peak from event timing as latency (Figures 3I and 3J), peak amplitude, and input amount (Figures S4A–S4C). To quantify discrepancies in the shapes of temporal filters, we calculated Pearson's correlation among the temporal filters (Figure S4G).

Clustering

We performed a statistical classification of the population of glutamatergic inputs [29] (Figure 2). First, we used a sparse principal component analysis (sPCA) to extract temporal features in response to a modulating flash based on the SpaSM toolbox on MATLAB [53]. Next, we fitted a Gaussian mixture model based on the expectation maximization algorithm using the MATLAB gmdistribution function to the dataset of detected sparse features. To determine the optimal number of clusters in the model, we calculated the Bayesian information criterion (BIC) score (Figure 2C) [32, 54]:

$$BIC = -2 * ln(L) + k * ln(n)$$

in which L is the log-likelihood of the model, k is the number of dimensions in the model, and n is the number of dataset.

To analyze similarity among the detected clusters, we performed a hierarchical clustering analysis based on a standard linkage algorithm using the MATLAB linkage function. As an input matrix for the clustering analysis to evaluate similarity in temporal features in response to the modulating flash (Figure S4), we used a feature matrix in which the mean of five features was calculated within the detected clusters in each row: peak latency, response decay, frequency and contrast sensitivity, and Pearson's correlation between stimulus profile and mean glutamate signal. To evaluate the similarity in glutamate signals to the modulating flash after blocking the GABA receptors, glycine receptors, and Na_V channels, we calculated the mean Pearson's correlation between the stimulus profile and glutamate signal (Figure 4).

Connectomic reconstruction

A previously published dataset acquired using SBEM was analyzed (retina k0725) [20]. Voxel dimensions were 13.2 × 13.2 × 26 nanometer (nm) (x, y, and z, respectively). Two ON DS cells fragments (ON DSGC1, ON DSGC2) were identified by tracing dendrites postsynaptic to previously reconstructed ON SACs. We sampled 93 (61) conventional synapses (non-ribbon type synapses) onto ON DSGC1 (ON DSGC2) and the presynaptic neurons were reconstructed. For ON DSGC1 (ON DSGC2), 71 (61) synapses were formed by SACs and 22 (0) were formed by GACs. We then annotated the ribbon type synapses (n = 263) onto the trees and reconstructed the

presynaptic bipolar cells (n = 124 total bipolar cells). The bipolar cells were subsequently assigned types (5i, 5o, 5t, or 7) based on their axonal depth profiles in the IPL and the ability to form mosaics with little overlap between neighboring cells of the same type.

We then estimated where the ON DS cell fragments would fit within a hypothetical 400 um diameter ON DS cell. We use the inferred null directions from the cells as well as the tendency for the daughter dendrites of a branch point to be oriented away from the soma to estimate the location of the fragments. Finally, we projected the synapse locations formed by GACs and bipolar cells onto the aligned preferred null axis to estimate the spatial distribution of synapses.

All analyses were performed by tracing skeletons and annotating synapses using the Knossos software package (https://knossostool.org/) [55].

Model simulation

To examine the impact of the spatial distribution of glutamatergic inputs on direction selectivity and speed preference, we established a computational model based on spatiotemporal linear receptive fields [28] (Figure 7A). The glutamatergic inputs in each ROI was described by the spatiotemporal convolution of the stimulus input,

$$i(x,y,t) = \int^{\tau} s(x,y,t-\tau)F(x,y,\tau)d\tau$$

where $i(x,y,t)$ is the output of the ROI at location (x,y), $s(x,y,t\text{-}\tau)$ is the stimulus input to the ROI, and $F(x,y,\tau)$ is the receptive field of the unit. The decay in outputs of the linear filter was modulated by an exponential function with double-decay constants,

$$i(x,y,T) = i(x,y,T) * (\exp(-t/\tau_1) - \exp(-t/\tau_2))$$

where T is the time after glutamate signal peaked in response to the stimulus and τ is the decay constant (Figures S7A and S7B).

The stimulus input was a moving bar (300 × 300 μm; 100, 150, 250, 300, 500, 800, 1600 μm/s). The simulated outputs from each ROI during the motion stimulus were sorted based on their location from the preferred side to null side (Figures 7B and 7C). The sorted outputs were summed, and the peak maximum value of the summed input was quantified as the model output to an ON DS cell. The model DSI was calculated by model outputs to the preferred and null direction (Figure 7H).

We created two additional models (Figure 7F): "shuffled" in which the spatial location of each glutamatergic input was shuffled, and "sustained" in which the G1 and G4 groups were changed to G2 and G5 groups, respectively. In the two models, the number of glutamatergic inputs was the same as for the delay-and-summate model. The 50 different shuffled models were created, and their model outputs were averaged.

The performance of these models (Figure 7I) was quantified by root mean square (RMS) error between model and measured DSI in EPSC amplitude,

$$RMS = \sqrt{1/N_v \sum_{vi} (Data_{vi} - Model_{vi})^2}$$

where v_i is velocity, $Data_{vi}$ is measured DSI at velocity v_i, $Model_{vi}$ is model DSI at velocity v_i, and N_v is number of velocity conditions.

QUANTIFICATION AND STATISTICAL ANALYSIS

Data analysis and statistical tests were performed by MATLAB 2017b (Mathworks). To fit the functions to our dataset, we used linear regression to estimate slopes and mean activation time in the spatiotemporal receptive field (Figures 1I–1K). To estimate optimal velocity in firings, we used Gaussian function (Figures 1J and S1H). Fitting of the Gaussian function was based on the least-square method in MATLAB. To fit the convex hull to the dendrites of the ON DS cells (Figure 5C), we binarized a z stacked image of dendrites. The binarized images were fitted by convhull function in MATLAB.

In Figure 1, 16 ON DS cells and 16 non-DS cells were used. Error bars in Figures 1C and 1E were SD. In Figures 2, 3, 4, and 5, 1175 ROIs from 6 ON DS cells were used. Error bars in Figures 2G–2I were SE. In Figures 3 and 4, 125 G1, 134 G2, 172 G3, 248 G4, 268 G5, and 165 G6 ROIs were used in SR/TPMPA application (Figures 3B–3D). 131 G1, 128 G2, 188 G3, 232 G4, 254 G5, and 172 G6 ROIs were used in strychnine application (Figures 3E–3H). 141 G1, 132 G2, 191 G3, 258 G4, 269 G5, and 182 G6 ROIs were used in TTX and TTX/SR/TPMPA application (Figures 3I and 3J). The Box-and-Whisker plots indicate the median, the interquartile range, and the minimum to maximum of the datasets. Measured statistics were described as mean ± SD in the texts. No statistical tests were used to predetermine sample sizes. The sample sizes in this study were similar or larger than those in previous publications [10, 12, 22, 25]. Data collection and analyses in this study were not carried out blind to the conditions of the experiments.

DATA AND CODE AVAILABILITY

The datasets and code generated during this study have not been deposited in a public repository due to the large file size but are available from the Lead Contact, Keisuke Yonehara (keisuke.yonehara@dandrite.au.dk), upon request.

Current Biology, Volume *29*

Supplemental Information

Spatiotemporally Asymmetric Excitation Supports Mammalian Retinal Motion Sensitivity

Akihiro Matsumoto, Kevin L. Briggman, and Keisuke Yonehara

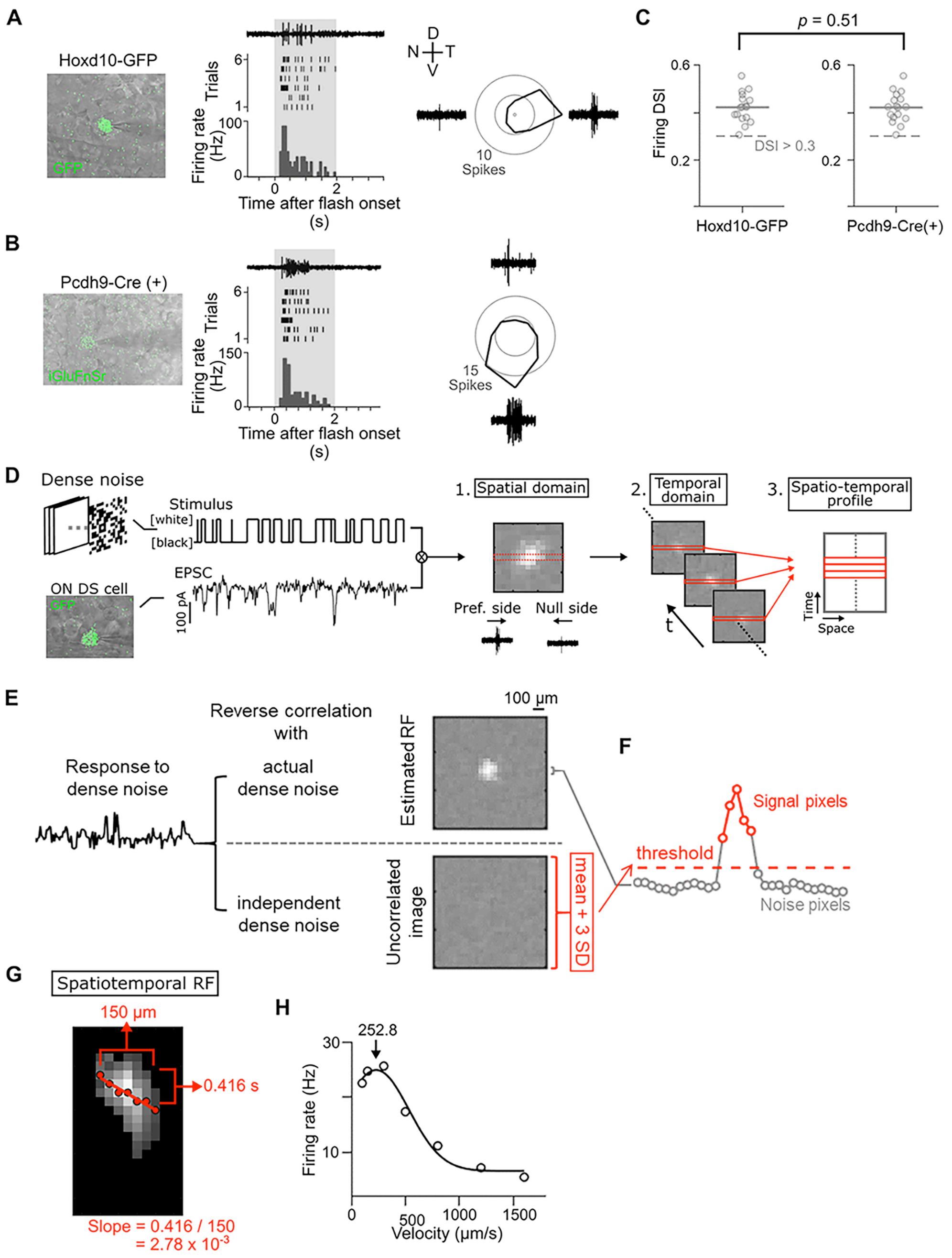

Figure S1. Recordings of Excitatory Inputs to Genetically-Labeled ON DS Cells, Related to Figure 1.

(A) Spike recording from an example Hoxd10-EGFP-labeled ganglion cell. It is known that all subtypes of ON DS cells are genetically labeled in Hoxd10-GFP mice. **(B)** Spike recording from

an example Pcdh9-Cre-labeled ganglion cell. It is known that ventrally-tuned ON DS cell subtype is genetically labeled in Pcdh9-Cre mice. **(C)** Firing DSI to a moving spot (300 µm in diameter, 150 µm/s) in Hoxd10-GFP and Pcdh9-Cre positive cells. All cells showed DS firings with more than 0.3 DSI. There were no significant differences in DSI between Hoxd10-GFP (17 cells) and Pcdh9-Cre positive cells (17 cells). $p = 0.51$, Mann-Whitney-Wilcoxon [MWW] test). **(D)** Top, reverse correlation using dense noise stimulus and evoked EPSC. Bottom, 1) spatial domain of RF. Red rectangle, a row including RF center along preferred-null axis. 2) temporal domain of RF at different synaptic delays was collected, and 3) aligned in spatiotemporal profile. **(E and F)** To make a criteria to determine a threshold for receptive field, we performed reverse correlation using response (EPSC or glutamate signal) to dense noise and independent dense noise ("uncorrelated image" in E). We calculated mean + 3 SD (red dashed line in F) of the uncorrelated image as a threshold to detect signal pixels in spatial receptive field (red circles in F). **(G)** Signal pixels in a thresholded spatiotemporal RF. Peak times in each spatial dimension (red circles) were fitted by linear regression (red line). The slope in peak time was calculated based on the fitted line (e.g. 0.418 s / 150 µm). **(H)** The optimal velocity in firings was calculated by a parameter (arrow) in the fitted Gaussian function.

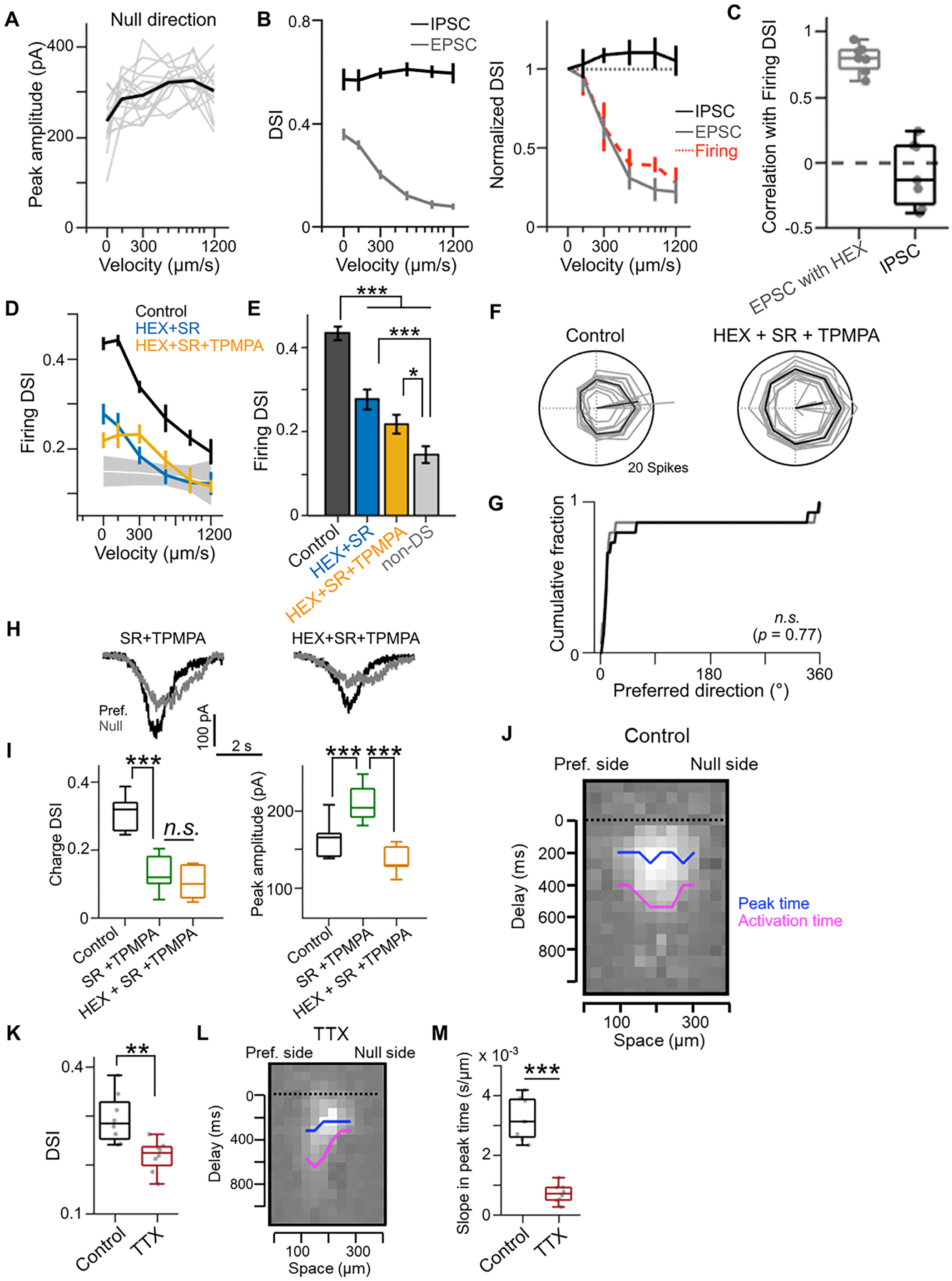

Figure S2. Electrophysiological Characterization of Inhibitory Inputs to ON DS Cells, Related to Figure 1.

(A) Inhibitory postsynaptic current (IPSC) amplitude recorded from ON DS cells as a function of stimulus velocity in null direction. Thin line, individual cell. Thick line, average. **(B)** Left, DSI calculated from peak amplitude of EPSC (gray) and IPSC (black). Right, DSI normalized to that

at 150 μm/s. 7 cells. Error bar, SE. **(C)** Correlation of ESPC and IPSC with firing DSI. **(D)** Relationship between firing DSI and motion velocity. Black, control, 16 ON DS cells. Blue, 2 μM hexamethonium (HEX) + 50 μM SR95531 (SR), 13 ON DS cells. Orange, HEX + SR + 100 μM TPMPA, 8 ON DS cells. Gray shaded, 95 % CI calculated from 16 non-DS cells. **(E)** Firing DSI to motion stimulus in 150 μm/s. Mann-Whitney-Wilcoxon test. **(F)** Directional tunings of firings in control (left) and HEX+SR+TPMPA (right). Gray, individual cells. Black, average. **(G)** Cumulative histogram of preferred direction (F). Gray, control. Black, HEX+SR+TPMPA. 12 cells. Kolmogorov-Smirnov test. **(H)** EPSCs to preferred- (black) and null- (gray) direction motion in SR+TPMPA (left) and in HEX+SR+TPMPA (right). **(I)** Charge DSI (left) and peak amplitude (right) of EPSC in control, SR+TPMPA (green) and HEX+SR+TPMPA (orange). 6 cells. Mann-Whitney-Wilcoxon test. **(J)** Spatiotemporal receptive field (stRF) for IPSC recorded from an ON DS cell. Detected peak timing (blue) and activation timing (magenta) were overlaid. Dotted black, timing of synaptic input. **(K)** DSI of EPSC to 150 μm/s motion in control (black) and in blocking of Na_V (TTX, 1 μM, dark red). 8 cells. One-tailed Wilcoxon signed-rank sum test. **(L)** Excitatory stRF in TTX. (M) Slope in peak time in control (black) and TTX (dark red). 8 cells. One-tailed Wilcoxon signed-rank sum test. *, $p < 0.05$. **, $p < 0.01$. ***, $p < 0.001$.

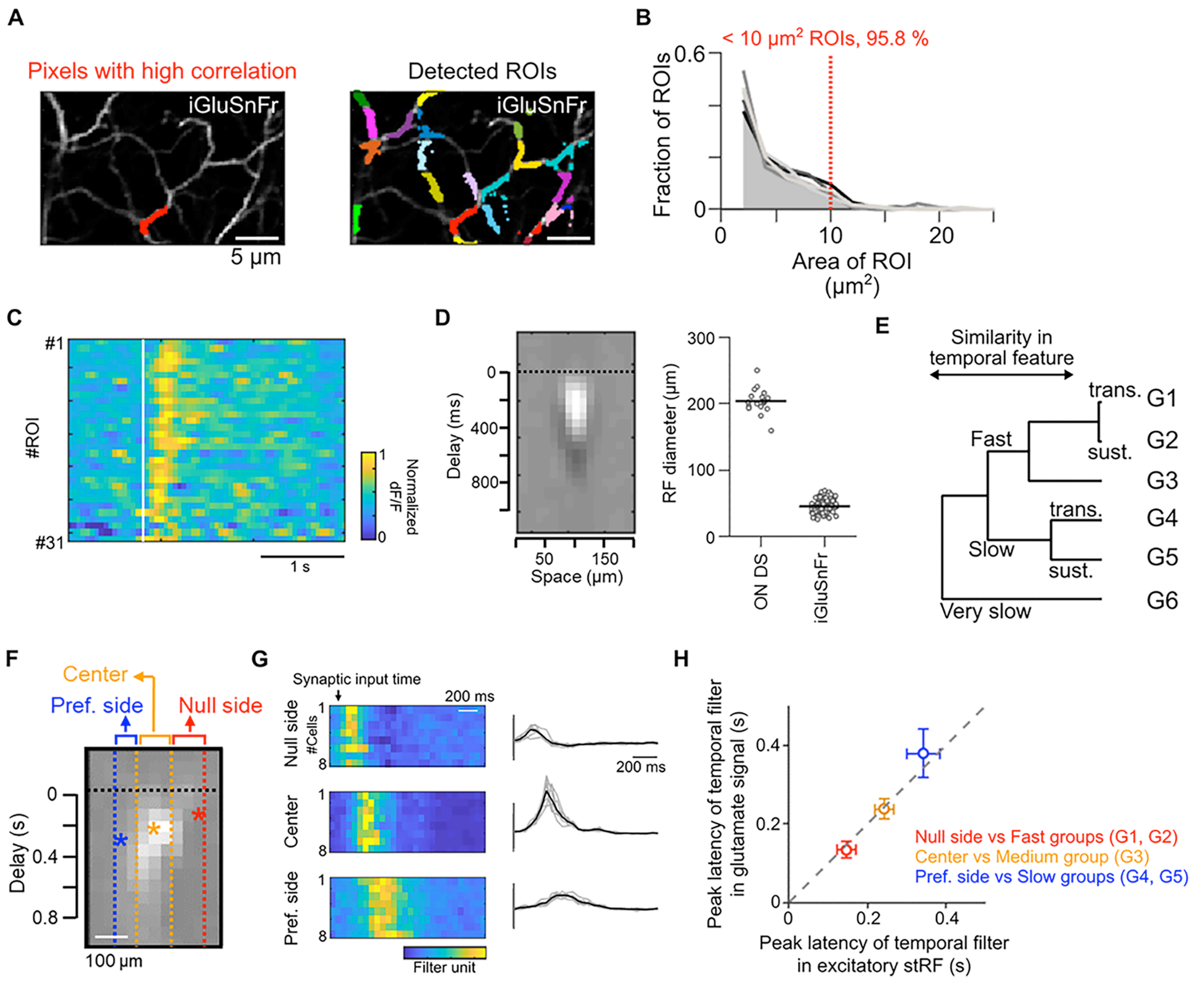

Figure S3. Determination of ROIs on the Dendrites of Genetically Labeled ON DS Cells for Glutamate Imaging, Related to Figure 2.

(A) Left, two-photon image of ON DS cell dendrites expressing iGluSnFr. Pixels showing high temporal correlation (> 0.4) in response to static flash stimulus were assigned to the same ROI (red pixels). Right, detected ROIs indicated by distinct colors. **(B)** Distribution of the area of ROIs. Based on the size of axonal boutons determined by a previous anatomical and glutamate imaging study (see ref. Franke et al., 2017), we set an upper limit of the area size to 10 μm^2. 95.8 % of responsive ROIs met this criterion. **(C)** Responses of ROIs detected in one field of view to a static flash for 2 s. White line, stimulus onset. **(D)** Left, an example stRF of glutamate signal. Spatiotemporally asymmetric structure is not obvious. Right, RF diameter of ON DS cells (17 cells) estimated by firings recorded by cell-attached recordings and spatial RF diameter of individual ROIs of glutamate signal (iGluSnFr, 128 ROIs). **(E)** Dendrogram estimated by

hierarchical clustering using temporal features: peak latency, decay, preferred frequency and contrast, correlation between glutamate signal and stimulus profile. **(F)** Schematic for the definition of the the preferred, center, and null side stRF (see also STAR Methods). We first detected three pixels: a center pixel (orange star) showing the maximum filter unit, and two pixels at the most preferred (blue star) and null (red star) side stRF. The center stRF (flanked by orange dotted lines) was defined as the three columns in a way that the central column includes the center pixel (orange star). The preferred and null side stRFs were defined as the columns between the blue or red star and the orange dotted line, respectively. **(G)** Left, heatmaps showing the calculated temporal filters at null side (top), center (middle), and preferred side (bottom) in 8 cells. Right, line plots of temporal filters in individual (gray) and the average (balck). Gray horizontal line, time of synaptic inputs. **(H)** Relationship between peak latency of temporal filters in excitatory stRF (red, null side; orange, center; blue, preferred side) and peak latency of temporal filter in glutamate signal (red, fast groups; orange, medium group; blue, slow groups). Mean ± SE in 8 cells.

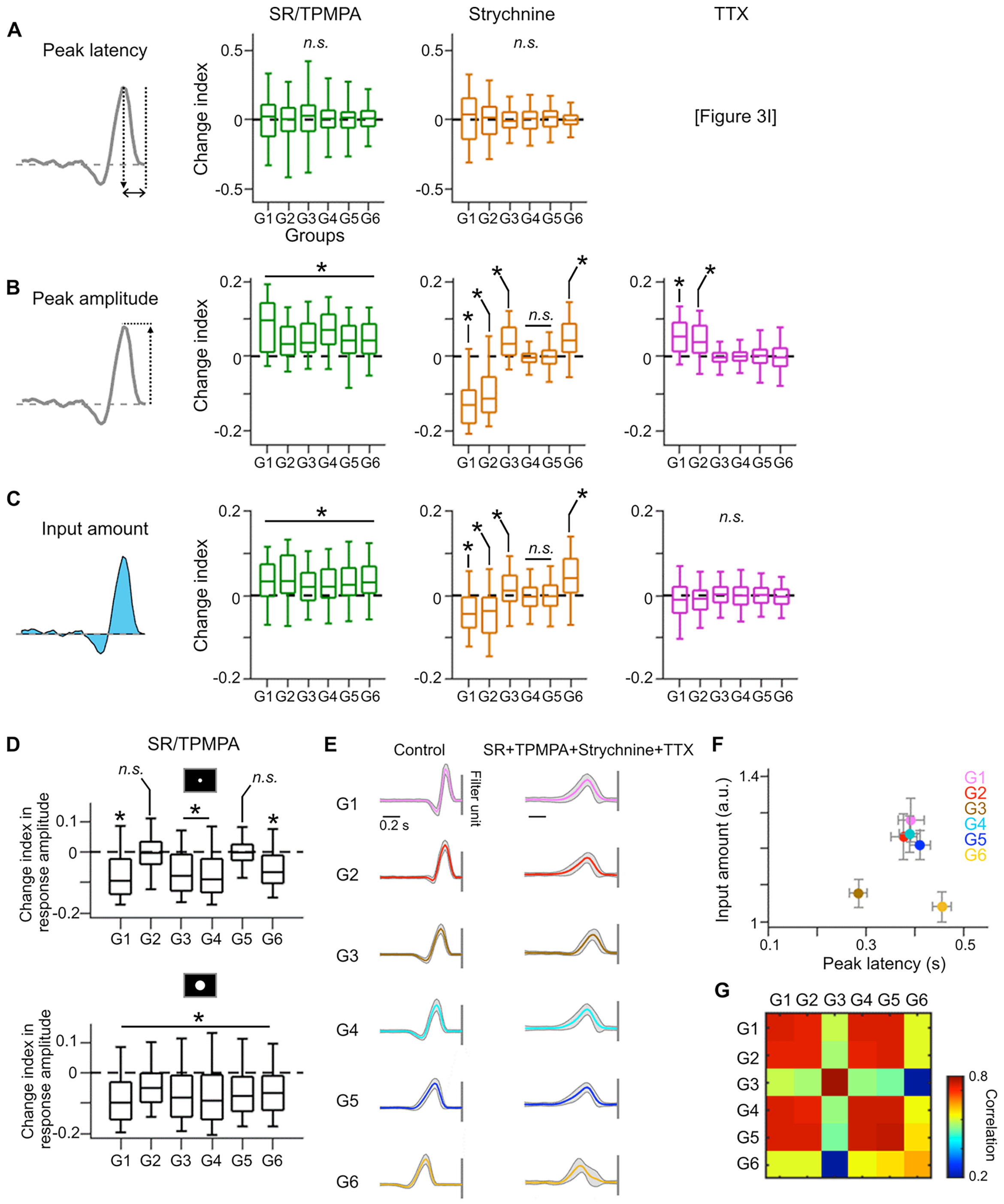

Figure S4. Different Effects of Pharmacological Blocking on Filter Properties of Glutamatergic Inputs, Related to Figures 3 and 4.

Effects of pharmacological blocking on temporal RFs were quantified by change index (see STAR Methods). Positive and negative values indicate increases and decreases in parameters by

application of blockers, respectively. Green, SR+TPMPA (50 and 100 μM). Gold, strychnine (1 μM). Purple, TTX (1 μM). **(A)** Change index in peak latency. Effects of TTX were shown in Figure 3I. **(B)** Change index in peak amplitude.**(C)** Change index in input amounts. **(D)** Change index in response amplitude of glutamate signals to small (top, 50 μm diameter) and large (bottom, 500 μm diameter) static flashing spot. **(E)** Temporal filter in control (left) and after the blocking of GABA, glycine, and acetylcholine receptors (right). Colored line, average. Gray shade, SD. **(F)** Peak latency and input amount of temporal filters in SR+TPMPA+Strychnine+HEX. Mean ± SE. **(G)** Mean correlation in the shape of temporal filters among the six groups. *, $p < 0.001$. One-tailed Wilcoxon signed-rank test.

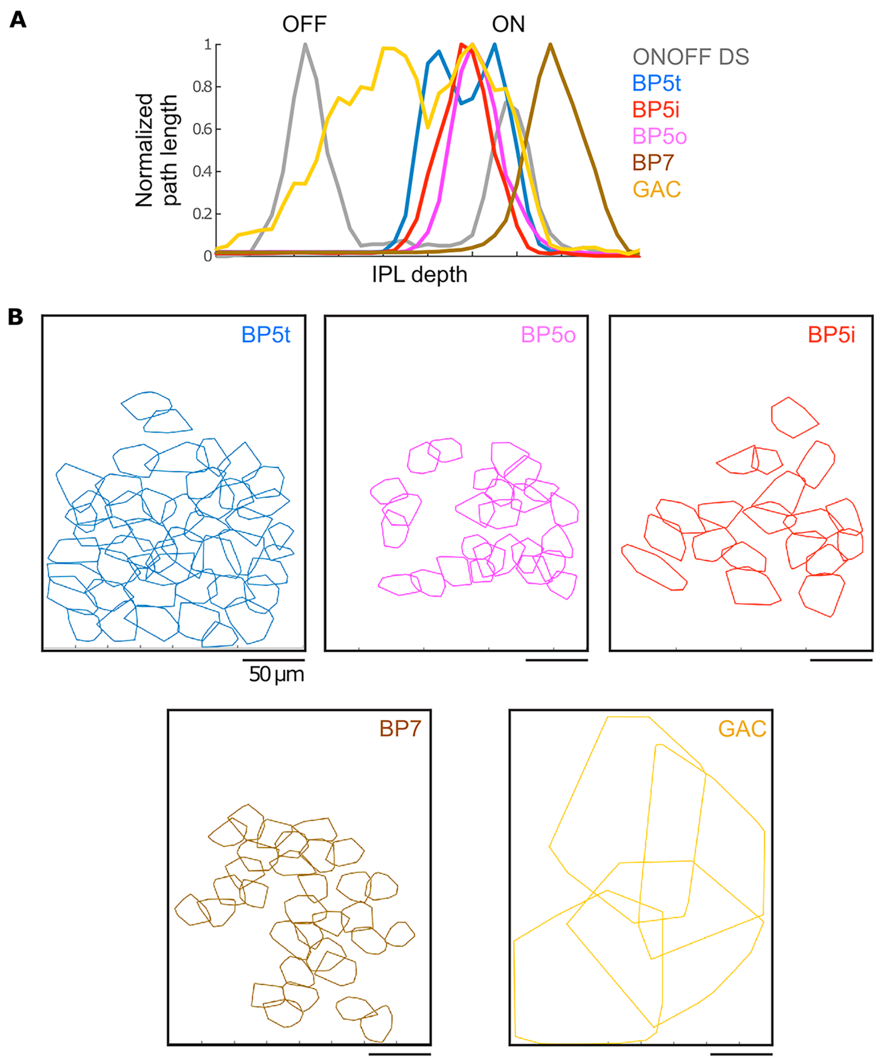

Figure S5. Classification of Excitatory Cell Types Presynaptic to ON DS Cells, Related to Figures 5 and 6.

(A) Stratification profiles of GACs and bipolar (BP) cells relative to OFF and ON DS cell bands in the IPL. **(B)** Mosaic of axonal convex hulls of BP types 5t, 5i, 5o, 7 and GACs. Scale bars: 50 µm.

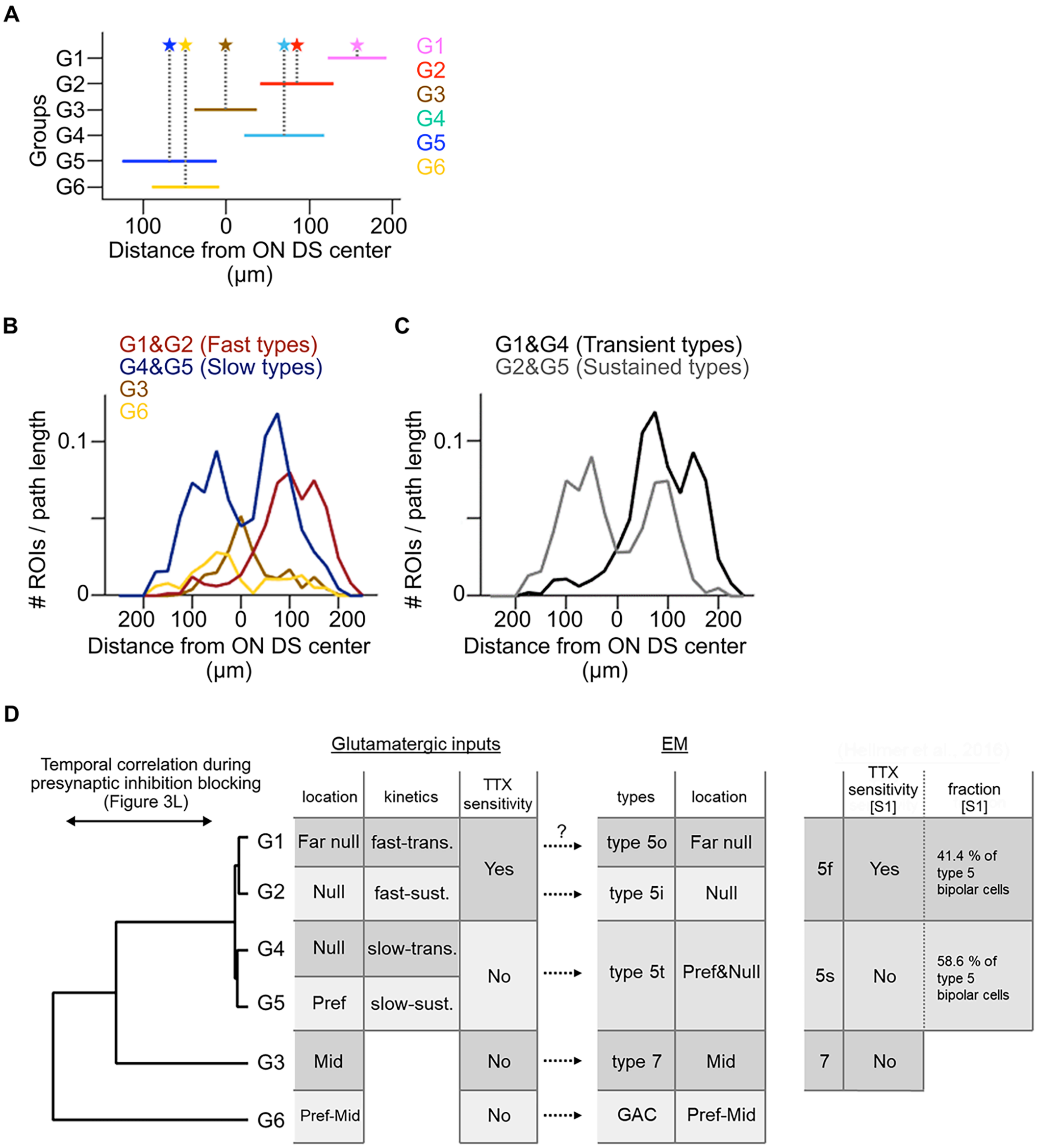

Figure S6. Spatial Distribution of Glutamatergic Input Groups, Related to Figures 5 and 6. **(A)** Summary of input location of the six glutamatergic groups. Each histogram of input location (Figure 5) was fitted by Gaussian distribution; mean (stars) and SD (horizontal line) in the fitted distribution were used to determine the input location. **(B and C)** Histograms of input location normalized by path length. Distributions of fast (G1 and G2) versus slow (G4 and G5) were summated in (B), and transient (G1 and G4) versus sustained (G2 and G5) were summated in (C). These distributions show that fast and transient inputs are biased to null side of ON DS cell dendrites. **(D)** Summaries for temporal correlation (dendrogram left), glutamatergic inputs (left

table), EM connectomics (center table), showing an estimation of the correspondence of the six groups in glutamate imaging to anatomical subtypes, and a reference of TTX sensitivity in anatomical subtypes from [S1] (right table).

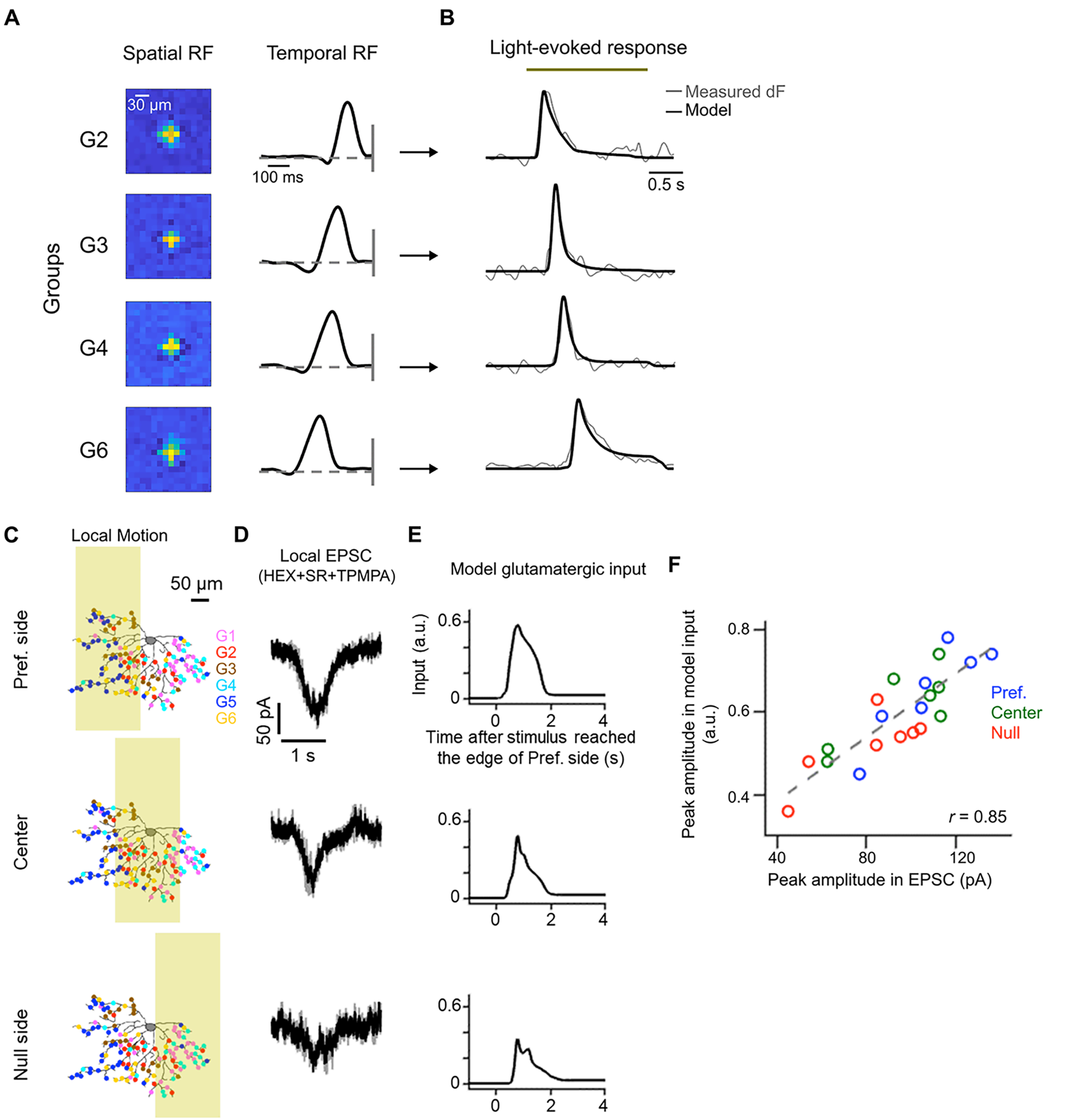

Figure S7. Linear Receptive Field Model to Simulate Light-Evoked Responses, Related to Figure 7.

(A) Spatial (left) and temporal (right) RFs in example 4 ROIs of G2, G3, G4, and G6 groups. Examples of G1 and G5 ROIs were shown in Figure 7A. **(B)** Simulated glutamatergic inputs to static flash (yellow horizontal bar) in the ROIs in (A). Gray, measured glutamatergic inputs. Black, modeled inputs. **(C)** Schematic for local motion (yellow band, 150 μm width × 400 μm length, 300 μm/s) at the preferred side (top), center (middle), and null side (bottom). **(D)** EPSCs to preferred-direction local motion at the preferred (top), center (middle), and null side (bottom). Gray, each trial. Black, average. **(E)** Model glutamatergic inputs simulated using ROIs which

were included in each local part (yellow bands in Figure S7C). **(F)** Relationship between peak amplitude in EPSC and model glutamatergic inputs at the preferred side (blue), center (green), and null side (red). 8 cells. $r = 0.85$. $p < 0.001$.

Supplemental Reference

S1. Hellmer, C.B., Zhou, Y., Fyk-Kolodziej, B., Hu, Z., and Ichinose, T. (2016). Morphological and physiological analysis of type-5 and other bipolar cells in the Mouse Retina. Neuroscience *315*, 246-258.
